# Association of coronary artery disease related single nucleotide-polymorphisms with extreme *Prakriti* types: Insights from a case control study

**DOI:** 10.1016/j.jaim.2026.101371

**Published:** 2026-07-08

**Authors:** Pamila Dua, Deep Shikha Punera, Dhwani Dholakia, Archana Vats, Shivam Pandey, Sandeep Seth, Mitali Mukerji, Subir Kumar Maulik, Bhavana Prasher, K.H. Reeta

**Affiliations:** a All India Institute of Medical Sciences, Ansari Nagar, New Delhi, 110029, India; b Institute of Genomics and Integrative Biology, CSIR, South Campus, Delhi, 110025, India; c Rajiv Gandhi Cancer Institute and Research Centre, Delhi, 110085, India; d Indian Institute of Technology, Jodhpur, 342030, India

**Keywords:** Coronary artery disease, Genomics, Ayurveda, Bioinformatics, Single nucleotide polymorphism, Susceptible SNPs, Indian population

## Abstract

**Background:**

Coronary artery disease has well-established genetic variants but identification of susceptible ones is needed. Ayurveda stratifies people phenotypically termed as “*Prakriti”* to assess the risk.

**Objectives:**

The present research was aimed to identify *Prakriti* stratified genetic risks for CAD.

**Material and methods:**

Initially, literature search was done using bioinformatics and manual-curation, to identify susceptible SNPs. Further, global-screening-array was performed to evaluate association of these polymorphisms in *Prakriti* stratified 200 CAD patients and 100 healthy controls. Thereafter, association of these polymorphisms with already done biochemical parameters and biomarkers was explored.

**Results:**

Bioinformatic approach resulted in 466 PMIDs reporting 694 SNPs associated with CAD. Further, manual search for only susceptible resulted in 255 susceptible SNPs across 134 candidate genes with 2.09 cumulative odds ratio in diverse populations. Among 255 SNPs, GSA analysis of isolated DNA samples of present cohort identified 5 SNPs (rs1544410, rs731236, rs1801133, rs7975232, and rs3825807) in VDR, MTHFR, ADAMTS7 genes in overall comparisons. While *Prakriti* stratification showed association of 4 SNPs in *Vata*, 8 SNPs in *Pitta* and 1 SNP in *Kapha Prakriti* group which were comparable with already documented biochemical parameters and biomarkers. Overall, stratification helped in assessing disease predisposition in specific *Prakriti* like *Vata* may be predisposed due to inflammatory pathways*, Pitta* due to Vitamin D deficiency and *Kapha* due to disturbed glycemic index.

**Conclusion:**

Overall, we could identify 255 SNPs as risk factors for CAD in diverse populations and 5 SNPs in Indian cohort. Further, integrating *Prakriti* stratification could help in identifying more précised risks for personalized CAD management.

## 1 Introduction

Coronary artery disease (CAD) is a condition in which the coronary arteries, which supply blood and oxygen to the heart muscle, become narrowed or blocked [1]. CAD is influenced by both genetic and environmental factors [2] and is a significant global health concern [3]. Understanding its causes, risk factors, and treatment options is essential for both healthcare professionals and the public to reduce its impact on heart health. The Framingham Heart Study has established many risk factors [4], nevertheless, the patients manifest symptoms even without any of the risk factors. Exploring an individual's risk for CAD at the time of birth is upcoming but it is not certain whether the subclinical disease will be expressed later or not. Hence, to do or not to do will always be a dilemma in cardiovascular research.

There are different guidelines available for risk stratification and management of CAD. For instance, the European Society of Cardiology has recommended risk stratification based on clinical assessment and diagnostic tests [5]. However, the complexities of invasive procedures and the economic burden on the patient cannot be ignored. The American Heart Association declared that an extensive risk factors assessment and management will lead to improved survival, decrease in further events as well as requirement of invasive procedures and ultimately a better quality of life [6]. Currently, “genomics” can serve as an epoch of individualized and targeted treatment [7]. Single nucleotide polymorphism (SNP), is the basic unit to evaluate the genetic variation between two individuals. SNPs may be exploited to ascertain disease-causing genes [8]. Researchers have explored various genes or polymorphisms associated with CAD. Studies have also been conducted in different populations exploring the relationship between specific gene variations/group of genes/SNPs/group of SNPs associated with CAD with variable outcomes showing both association as well as non-association with CAD; and even if associated, the variation may either be susceptible or protective towards CAD [9,10]. The studies also aimed to demonstrate the associated polymorphisms and the type of association [11]. Therefore, even a large number of polymorphisms associated with CAD documented in literature but in the era for preventive and personalized medicine, knowing susceptible polymorphisms and that too, population specific may be a big milestone towards this strategy. To the best of our knowledge there is hardly any study focusing only on susceptible polymorphisms for CAD. Therefore, our literature search aimed to isolate only susceptible polymorphisms, which are the genetic variations that make an individual more predisposed towards a specific pathological condition or disease. The odds ratio (OR) serves as a statistical metric in epidemiology and gauges the degree of association between two variables and OR > 1 is a feature for susceptibility to the disease [12]. Researchers utilize this scale to assess both the strength and direction of connections to estimate the potential risk factors that may influence a particular outcome. Therefore, the present study was primarily aimed to identify genetic variants associated with susceptibility to CAD with OR > 1. To achieve this, an extensive and thorough literature search was carried out, seeking to compile a comprehensive database of relevant studies and their corresponding SNPs. There are only a handful of studies showing such associations in CAD and only a little is known in Indian population.

Ayurveda, the ancient science of life, aims at both the prevention of disease and the promotion of overall well-being [13]. Much like modern genomics, Ayurveda offers valuable clinical insights aligned with the principles of P4 medicine—predictive, preventive, personalized, and participatory. A distinctive feature of this traditional system is the concept of *Prakriti*, which classifies individuals based on their unique physical and psychological constitution, accounting for inter-individual variability [14]. This phenotypic classification enables the stratification of individuals to assess their personalized risk for disease predisposition and to guide tailored therapeutic interventions. *Prakriti* is primarily categorized into three dominant types like *Vata, Pitta*, and *Kapha* and their combinations make a total of seven subtypes as described in classical texts. Biochemical and immunological studies have identified that these *Prakriti* types exhibit differences in metabolic rates, inflammatory markers, and response to nutritional interventions. This further validates the physiological relevance of Prakriti classification [15].

In the current era, there is growing emphasis on holistic and integrative approaches to health that combine diverse medical systems to improve human well-being. In this context, the present study was conducted through a collaborative effort between the Departments of Pharmacology and Cardiology at All India Institute of Medical Sciences, New Delhi and the Tri-Sutra Unit of Institute of Genomics and Integrative Biology, New Delhi. This study aimed to identify risk factors in terms of biochemical parameters, biomarkers and susceptible genetic polymorphisms associated with CAD in an Indian cohort. The comparisons between the different biochemical parameters and biomarkers in predominant *Prakriti* types of this cohort have been published earlier [16]. Furthermore, personalized CAD risks were explored through an integrative approach combining principles of Ayurveda and genomics in the present study.

## 2 Methods

The study was conducted in two distinct phases: Phase I and Phase II.

**Phase I:** Bioinformatics-assisted literature mining and manual curation to identify susceptible SNPs associated with CAD.
**Phase II:** To evaluate association of these polymorphisms in *Prakrit*i-stratified cohort of CAD patients and healthy controls using Global Screening Array (GSA).

### 2.1 Phase I

The first phase comprised a comprehensive literature search to identify relevant studies and SNPs associated with CAD. The process started with the search for studies and associated SNPs for CAD using bioinformatics. First, to get evidence of gene variants or SNPs associated with CAD, studies were searched from PubMed by using different terms like Coronary artery disease, Gene, Variants, SNPs. Relevant Medical Subjects Headings (MeSH) terms or key words were extracted. The identified variants were associated in different ways with CAD. For instance, the associations were not only susceptible or protective, but also showed diverse associations including linkage with y chromosome/smoking habit/familial hypercholesteremia/diabetes mellitus/hypothyroidism/chronic obstructive pulmonary disease, etc. A sequence of machine learning processes in bioinformatics like PubMed parser [17] and NLP techniques [18] were applied to approximately 28 million PubMed abstracts to organize and get a structured information on SNPs, population, and diseases. Further, python scripts [19] were utilized to extract and curate disease-related data, filter out false positives, and to categorize into hierarchical groups using named entity recognition (NER) [20] algorithms to find PMIDs and SNPs associated with CAD.

Further, we narrowed our focus through OR to search exclusively for susceptible SNPs associated with CAD within the mined literature retrieved from studies including diverse global populations using manual approach. It was done using the retrieved PMIDS and SNPs in CAD. The studies were read thoroughly to screen the polymorphisms susceptible to CAD. The studies which fulfilled the inclusion/exclusion criteria were included. Publications with case and control groups evaluating the association of polymorphisms and the risk of CAD as the main outcome, studies in which polymorphisms expressed with an OR > 1 and 95% confidence interval (CI) and articles published in English language were included. The studies not related to CAD, duplicate studies, letters, reviews, and animal experiments were excluded. The list of susceptible polymorphisms with OR > 1 including diverse global population was prepared.

### 2.2 Phase II

Phase II included screening and recruitment of the participants of Indian cohort including both CAD patients and healthy controls. Before recruitment of the participants, Institute Ethics Committee approval (IEC-510/05.10.2018, RP-40/2018) was obtained and study was prospectively registered in Clinical Trial Registry-India (CTRI/2019/01/016866 on 03.01.2019).

Further, blood sample collection for isolation of DNA followed by GSA and analysis was done. The analysis was done to identify the expression of pool of polymorphisms retrieved from literature search in our studied population and then it was explored how these genomic expressions are associated with earlier done biochemical parameters and biomarkers.

#### 2.2.1 Study design

The present study was an observational study. The recruitment of participants was conducted in a single tertiary care center. The study was carried out according to ethical guidelines for biomedical research on human subjects (2017) [21]. Signed written informed consent was obtained from all the participants of the study. All procedures performed in this study were in accordance with the ethical standards of the institutional ethics committee and with the 1964 Helsinki Declaration and its later amendments or comparable ethical guidelines [22].

#### 2.2.2 Sample size

The present study is continuation part of earlier published study in which sample size of 200 CAD patients and 100 healthy controls was selected [16].

#### 2.2.3 Screening

The selection of 200 CAD patients from the 852 screened individuals and 100 healthy controls from 178 volunteers was based on stringent inclusion and exclusion criteria. All patients had a confirmed diagnosis of CAD, established by a consultant cardiologist through coronary angiography, ECG-confirmed, or echocardiographic evidence of CAD.

#### 2.2 d Recruitment

The first recruitment was done in the month of May, 2019. The recruitment of all participants of both groups was completed by month of May, 2023. Participants in group I (CAD patients) were screened from Cardiology OPD and healthy controls (group II) were screened from the attendants of the patients and other healthy volunteers working in the Institute. The selected patients were of stable CAD, aged between 18 and 80 years, with no acute myocardial infarction in the past 3 months, no severe renal or hepatic dysfunction (defined as enzyme levels more than 3 times the upper normal limit), and who were not on any complementary/traditional medicine. Whereas, healthy controls were selected based on clinical evaluation, absence of any known chronic illnesses, metabolic syndrome, or intake of medications influencing cardiovascular risk and all biochemical parameters within the normal range.

#### 2.2.5 Sample collection

Peripheral blood samples of all recruited participant were collected in labeled vacutainers as per standard guidelines.

### 2.3 *Prakriti* assessment

The data analysis to identify Prakriti was executed through detailed history and examination done by the physician followed by a software-based analysis. In *Prakriti* assessment process, physician conducts a thorough examination, collecting detailed information on the individual's demographic, anatomical, physiological, behavioral, and emotional characteristics. A validated questionnaire designed to capture data across various domains, including health features and patterns related to *Vata, Pitta*, and *Kapha* doshas to assess *Prakriti* was used. The tool has been employed in previous models for *Prakriti* assessment [23] and finally, the gathered data was used in a specialized software that employs artificial intelligence and machine learning algorithms. These algorithms analyze the data to determine the predominant dosha, effectively identifying the individual's *Prakriti*.

### 2.4 DNA isolation and GSA

**Fig. 1:**
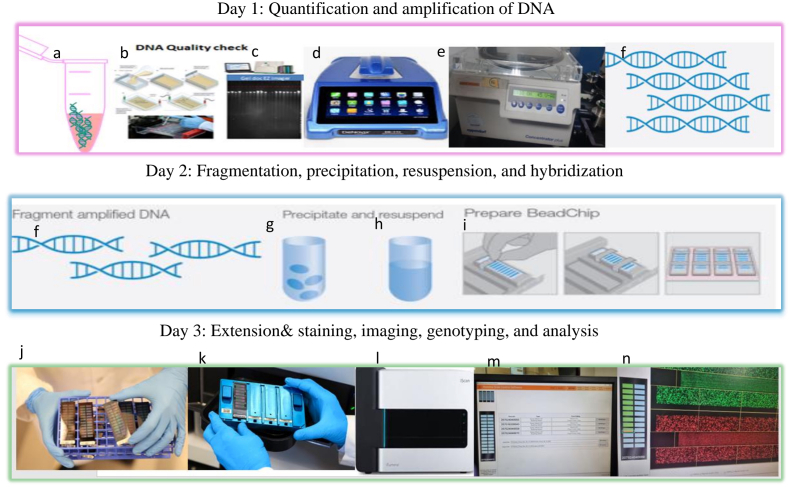
Demonstrating step by step three days procedure of GSA, first day starts from DNA isolation to quality check by agarose-gel electrophoresis followed by quantification through nanodrop spectrophotometer, after that preparation of DNA for required concentration by dilution or concentrator. Second day procedure started fragment amplification followed by precipitation and re-suspension and then bead chip preparation to the elution of the chip. Third day involved extension, staining, hybridization followed by imaging, genotyping, and analysis. Further, details are like a. Genomic DNA is isolated using standard kits from peripheral blood sample **b**. Gel electrophoresis method **c.** Gel-Doc instrument and results of gel-run **d.** Nono-drop instrument for DNA quantification **e**. DNA concentrator for processing the samples with low concentration **f**. Absolute DNA ready for GSA **g**. Amplification to generate sufficient material for array hybridization. **h**. DNA precipitation and washing done to remove enzymes and salts. **i.** DNA is resuspended in hybridization buffer. **j**. Samples are loaded onto the Illumina GSA Bead-Chip and DNA fragments hybridize overnight to locus-specific oligonucleotide probes attached to beads **k.** A single-base extension reaction occurs at the SNP site. **l.** Excess reagents are washed off the BeadChip and fluorescently labeled nucleotides are incorporated based on allele identity. **m.** The chip is scanned using the Illumina iScan System. **n.** Results of genotyping from machine in samples **o.** Fluorescent signals: detected and recorded.

Identification of extracted pool of susceptible SNPs was done in a set of Indian population including 200 CAD patients and 100 healthy controls. DNA isolation from blood sample was performed following the appropriate protocol using QIA-amp DNA Blood Micro Kits (QIAGEN) [24]. Further, GSA was executed from isolated DNA samples and genotyping was done on the Illumina I-scan system using the Infinium Global Screening Array-24 v1.0 Bead Chip (6,42,824 genome wide SNP probes) and Global Screening Array-24 v3.0 Bead Chip (6,54,027 genome wide SNP probes) [25]. The process involved the steps like quantification and amplification of DNA on the first day, followed by fragmentation, precipitation, resuspension, and hybridization on the second day. On the third day, extension and imaging of bead chips were performed, followed by extraction of the genotype data from intensity data files. For GSA sufficient and good quality DNA was used and the required concentration for DNA was 50 ng/μL. All the samples were prepared accordingly, samples with high concentration were diluted and similarly with low concentration were dried to get optimal concentration for performance. Thereafter, the sample passed gene call rate ≥0.95 to get included for analysis. Figure-1 is explaining about the *DNA isolation and execution of GSA.*

### 2.5 GSA analysis

As the GSA was performed in two batches using two different versions of the chip sets, the data were preprocessed separately for both and then was merged later. The raw data of the samples with the genotype call rate ≥0.95 were loaded into Genome Studio Software v2.0 in two batches [26]. A total of 6,01,700 SNPs in the first batch and 6,18,404 SNPs in the second batch were selected with GenTrain score ≥0.7 and cluster separation/cluster-based GSA ≥0.3. After this, the data was run in PLINK 1.9 [27] for quality control (QC) and analysis.

Using PLINK, samples and variants with 10% missing values were filtered out, which removed 18237 SNPs from the first batch and 22740 SNPs from the second batch while no samples were excluded. Then the Hardy Weinberg Equilibrium (HWE) exact test was applied to filter out the SNPs deviating significantly with p values < 10^−6^. A total of 9210 SNPs from the first batch and nil from the second batch were removed in this step. To concentrate on the common variants, minor allele threshold of 0.05 was applied. This removed 285982 SNPs from the first batch and 295122 from the second batch. Thus, 288271 variants in the first batch and 300542 variants in the second batch passed the filters and QC.

Then, the two datasets generated after QC were merged. To do that, first the list of common SNPs in both datasets was generated. 250095 SNPs were found to be common in both sets. The data for only these common SNPs were extracted from each set of files in PLINK and the files thus generated were merged.

The case control allelic test report was then generated. Fischer's exact test was performed to generate p values. Genomic control correction [28] was applied and genomic control corrected p values were generated. Similarly, the report was generated for three predominant *Prakriti* stratified groups also.

### 2.6 Association with already done biochemical parameters and biomarkers

**Fig. 2:**
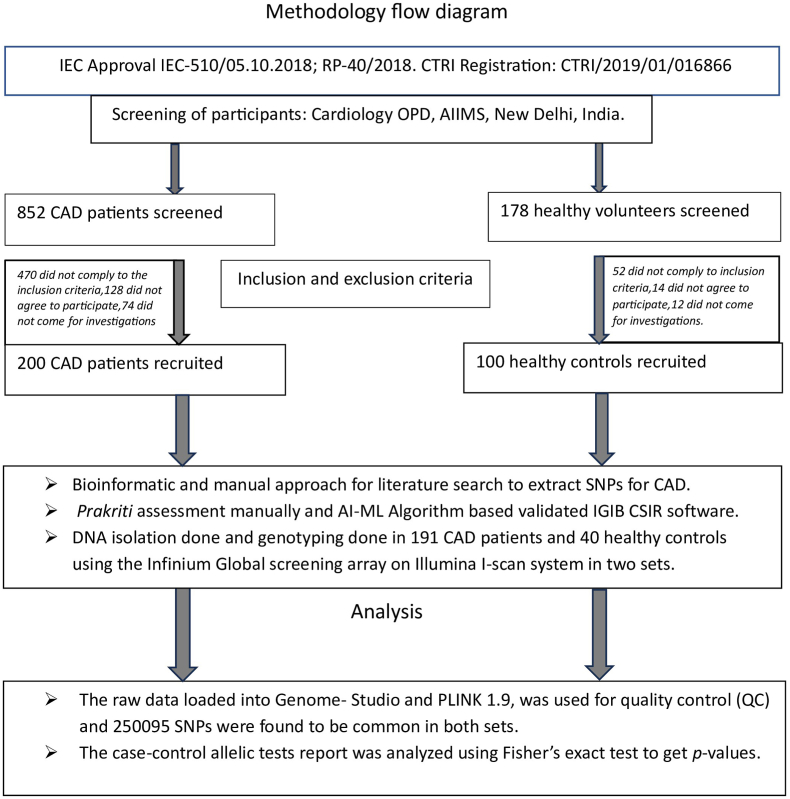
Methodology flow diagram.

Once the genomic results for overall CAD patients and healthy controls and their *Prakriti* stratified groups were analyzed, it was tried to find the association between these genomics and already done biochemical parameter and biomarkers which included a comprehensive set of tests to incorporate various aspects of CAD pathophysiology like hemogram with differential cell count, monocyte to lymphocyte ratio (MLR), lipid profile, fasting blood sugar (FBS), glycosylated hemoglobin (HbA1c), liver function test (LFT)], kidney function test (KFT), C-reactive protein (CRP), interleukin-6 (IL- 6), proprotein convertase subtilisin/kexin type-9 (PCSK9), lipoprotein-associated phosphor-lipase A2 (LpPLA2), malondialdehyde (MDA), catalase, glutathione (GSH), superoxide dismutase (SOD), and N-terminal pro B-type natriuretic peptide (NT-pro BNP) were performed. The complete data of these parameters have been published in detail [28]. The entire methodology is demonstrated in Fig. 2.

## 3 Results

### 3.1 A) phase I

#### 3.1.1 Determination of susceptible polymorphisms for CAD

Literature search for studies and associated SNPs for CAD could retrieve 466 PMIDs with 694 SNPs. Data and text search of all studies and segregation based on susceptibility with OR>1 was prepared. 347 polymorphisms from 137 studies were included for analysis. The data were analyzed to get cumulative OR. The details of the data are presented in **Supplementary file 1.**

#### 3.1.2 B) Ontological details

Further, after removing the duplicates polymorphisms, 255 SNPs were retrieved and the list of 255 susceptible SNPs is provided in **Supplementary file 2.** 255 polymorphisms identified were associated with 134 genes. Maximum polymorphisms were in intronic region, followed by exonic, intergenic, upstream, 3′-UTR, ncRNA-intronic, 5′-UTR, downstream, upstream, upstream-downstream, ncRNA-exonic and one was synonymous.

#### 3.1.3 C) cumulative odds ratio

Cumulative OR was calculated using estimated OR, lower limit and the standard error. In case the same polymorphism was observed in different population groups, then all the associations were recorded to calculate cumulative OR. Further, the highest ORs was selected for analysis if there were more than one genotype associated with the same SNP. The analysis was done using RevMan 5.4 statistical software. The results are presented in **Supplementary file 3**. The cumulative OR of each allele was estimated with a random effect model by generating the cumulative 95% CI using the generic inverse variance method. Data of all 347 SNPs were analyzed to get the estimated overall cumulative OR, which was 2.09 (1.424 to 2.674).

### 3.2 Phase II

#### 3.2.1 *Prakriti* assessment results

Overall, the population in two groups included 200 CAD patients and 100 healthy controls were stratified in different *Prakriti* groups. In the CAD group, 39.5% of patients were *Kapha*-predominant, 38% were *Vata-*predominant, and 21% were *Pitta*-predominant. Among healthy controls, 42% were *Vata*-predominant, 28% were *Pitta-*predominant, and 29% were *Kapha*-predominant (see Table 1). There was also a small *Sama* (mixed) *Prakriti* subgroup comprising three CAD patients and one healthy control. Therefore, this group was omitted from further analysis due to its limited size (Table 1: Demonstrating the distribution in Prakriti predominant groups).

**Table 1:** Distribution of all participants in different *Prakriti* groups.

Predominant *Prakriti*	CAD patients (n = 200)	Healthy controls (n = 100)
***Vata* participants**	76 (38%)	42 (42%)
***Pitta* participants**	42 (21%)	28 (28%)
***Kapha* participants**	79 (39.5%)	29 (29%)
***Sama (Mix)* participants**	3 (1.5%)	1 (1%)

#### 3.2.2 GSA results is overall case- control groups

Comparison of GSA in CAD patients and healthy controls showed an association of 12446 SNPs with p < 0.05. Significant minor allele frequency (MAF) differences were observed in 197 SNPs between CAD patients and healthy controls with genomic control corrected p < 0.001. Amongst list of 255 susceptible SNPs, 92 variants were present in our GSA array, of which, 5 polymorphisms showed significant association in our population set. The polymorphisms representing noteworthy association with CAD were rs154410, rs7975232 and rs731236 of *VDR* gene at chromosome 12, rs3825807 of *ADAMTS7* gene at chromosome 15 and rs1801133 of *MTHFR* gene at chromosome 1 (Table 2).

**Table 2:** Details of 5 polymorphisms, their location and allele frequencies which showed significant difference in CAD patients and healthy controls in present cohort.

SNP	Chromo some	Gene	Base pairs	Reference allele	Frequency Alt. allele in cases F_A	Frequency Alt. allele in controls F_U	Alternate allele	Chi-square	p value	OR
rs1544410	12	VDR	48239835	A	0.4851	0.2586	G	9.41	0.0022	2.70
rs3825807	15	ADAMTS7	79089111	G	0.2626	0.4483	A	7.298	0.0069	0.44
rs7975232	12	VDR	48238837	C	0.3861	0.569	A	6.156	0.0131	0.48
rs1801133	1	MTHFR	11856378	A	0.1535	0.2963	G	5.799	0.016	0.43
rs731236	12	VDR	48238757	G	0.3614	0.1964	A	5.433	0.0198	2.32

Further, functional annotation of significantly associated 3 genes expressed in our population subset to investigate the biological significance was performed using the DAVID Functional Annotation Tool [29]. The gene *ADAMTS7* demonstrated associated biological processes like extracellular matrix organization, proteolysis, and proteoglycan metabolism. The cellular components were in the extracellular region and matrix [30]. The *MTHFR* gene expressed biological processes including methionine biosynthesis, homocysteine metabolism, and response to vitamin B2 and folic acid [31]. The *VDR* gene was associated with the biological processes like calcium ion transport, intracellular calcium homeostasis, vitamin D receptor signaling, and transcriptional regulation by RNA polymerase II. Further, cellular components comprised nucleus, chromatin, and receptor complexes [32,33]. These findings align with the known roles of these genes in CAD-related processes such as vascular remodeling, endothelial dysfunction, and mineral metabolism [34].

#### 3.2.3 GSA results in prakriti stratified case- control groups

Comparison of GSA in *Vata* group of CAD patients and *Vata* healthy controls for 255 associated susceptible SNPs implicated the significant association of 4 polymorphisms rs1544410, rs3025000, rs1883832, rs7566605 from 3 genes *VDR, VEGFA, CD40* and one at intra-genic location of *CCD93:INSGI2* (Table 3) Comparison of GSA in *Pitta* group of CAD patients and healthy controls for association of 255 susceptible SNPs demonstrated significant association of 8 polymorphisms named rs1544410, and rs7975232 from *VDR* gene of chromosome 12.and ×861539 at *XRCC3* gene of chromosome 14. Further, rs1800796 at *IL6* gene of chromosome 7, rs1883832 at CD40 of chromosome 20, rs2066865 at *FGG* gene of chromosome 4, rs7521023 at *CASQ* gene1of chromosome 2 and rs5882 at *CETP* gene of chromosome 16 (Table 3). Comparison of GSA in stratified *Kapha* group of CAD patients and healthy controls for 255 associated susceptible SNPs portrayed the association of rs1801133 of *MTHFR* gene at chromosome 1 (Table 3).

**Table 3:** Significant susceptible polymorphisms in *Vata, Pitta and Kapha Prakrti* CAD patients and *Vata, Pitta and Kapha Prakrti* healthy controls.

Chr.	SNP	Gene	BP	A1	F_A	F_U	A2	Chi Sq	p-value	OR
***Vata* CAD patients Vs All healthy controls**										
12	rs1544410	VDR	48239835	A	0.46	0.2586	G	4.775	0.02888	2.442
6	rs3025000	VEGFA	43746169	A	0.36	0.1552	G	6.008	0.01424	3.062
20	rs1883832	CD40	44746982	A	0.32	0.1552	G	4.101	0.04286	2.562
2	rs7566605	CCDC93; INSIG2	118836025	C	0.16	0.3793	G	6.438	0.01117	0.3117
***Pitta* CAD patients Vs All healthy controls**										
12	rs1544410	VDR	48239835	A	0.4808	0.2586	G	5.847	0.0156	2.654
12	rs7975232	VDR	48238837	C	0.2885	0.569	A	8.771	0.00306	0.3071
14	rs861539	XRCC3	104165753	A	0.1538	0.3621	G	6.123	0.01334	0.3203
7	rs1800796	IL6	22766246	G	0.4231	0.2414	C	4.111	0.0426	2.305
20	rs1883832	CD40	44746982	A	0.3462	0.1552	G	5.4	0.02014	2.882
4	rs2066865	FGG	155525276	A	0.3846	0.1897	G	5.149	0.02326	2.67
1	rs7521023	CASQ2	116243380	G	0.5	0.2931	A	4.929	0.0264	2.412
16	rs5882	CETP	57016092	G	0.3654	0.569	A	4.559	0.03275	0.4362
***Kapha* CAD patients Vs All healthy controls**										
1	rs1801133	MTHFR	11856378	A	0.06897	0.2963	G	9.852	0.0016	0.1759

#### 3.2 d Biochemical parameters and biomarkers

Biochemical parameters and biomarkers done in the same group of CAD patients and healthy controls. The table-4 is demonstrating the trend and comparison of the biochemical parameters and biomarkers in overall CAD patients and healthy controls further, in *Prakriti* stratified groups. (Table 4). The results data of biochemical and biomarkers [22] were used to find out their association with the genomic expressions of the current study.

**Table 4:** Biochemical parameters and biomarkers: results from previously published paper. ↑↑ means higher in CAD patients as compare to healthy controls and ↓↓means lower in CAD patients as compare to healthy controls. ∗/#/$ shows significant difference in specific Prakriti classified groups (V/P/K) compared to all healthy controls.

Parameters	Over all CAD patients and healthy controls	*Vata* CAD patients and healthy controls	*Pitta* CAD patients and healthy controls	*Kapha* CAD patients and healthy controls	*Within VPK CAD patients p value*	Reference range
**Total leucocyte counts**	↑↑	↑↑∗	↑↑ #	↑↑$	0.321	4000-11000 cells/μL
**Neutrophils**	↑↑	↑↑∗	↑↑#	No Difference	0.435	40-70% of WBC
**Lymphocytes**	↓↓	↓↓∗	↓↓#	↓↓$	0.775	20-40% of WBC
**Platelet count**	↓↓	↓↓∗	No Difference #	↓↓ $	0.05	150,000–450,000 cells/μL
**Monocytes lymphocyte ratio**	↑↑	↑↑∗	No Difference	No Difference	0.526	0.10-0.30 ratio
**Total cholesterol**	↓↓	↓↓∗	↓↓#	↓↓	0.002	<200 (desirable) mg/dL
**LDL**	↓↓	↓↓∗	↓↓#	↓↓	0.04	<100 (optimal) mg/dL
**HDL**	↓↓	↓↓∗	No Difference	No Difference	0.306	>40 (men), >50 (women) mg/dL
**Blood urea**	↑↑	↑↑∗	↑↑#	↑↑$	0.432	15 – 40 mg/dL
**Creatinine**	↑↑	↑↑∗	No Difference#	↑↑$	0.018	0.6 – 1.2 mg/dL
**Uric acid**	↑↑	↑↑∗	No Difference	↑↑$	0.236	3.4 – 7.0 (men) 2.4 – 6.0 (women) mg/dL
**Fasting blood sugar**	↑↑	No Difference ∗	No Difference #	↑↑ $	0.899	70-110 mg/dL
**HbA1c**	↑↑	↑↑∗	↑↑#	↑↑$	0.325	4.0-5.6%
**S. alkaline phosphatase**	No Difference	No Difference	↑↑#	No Difference	0.028	44-147 U/L
**IL-6**	No Difference	No Difference∗	No Difference	No Difference	0.001	<7 pg/mL
**PCSK9**	No Difference	No Difference∗	No Difference	No Difference$	0.008	100-1000; typical ∼200–400) ng/mL
**LpPLA2**	No Difference	Difference∗	No Difference	No Difference	0.007	<200 ng/mL
**NT-pro BNP**	↑↑	↑↑∗	↑↑#	↑↑$	0.047	<125 (<75 years) pg/mL
**MDA**	↑↑	No Difference∗	No Difference	↑↑$	0.129	0.5 – 2.5 μmol/L
**Catalase**	No Difference	No Difference	↑↑	No Difference $	0.062	20 – 60 U/mL

#### 3.2.5 Vitamin D deficiency in overall/P*rakriti* stratified groups

Since the three polymorphisms among 5 in overall CAD patients and healthy controls, were associated with VDR gene and vitamin D estimation was not included in the original study methodology. However, some of the patients and controls had undergone routine vitamin D testing as part of their standard clinical evaluation during screening in the hospital, since this data was available in the biochemical records and less than 20 ng/mL was taken as moderate deficiency. Further, a post hoc exploratory analysis of serum vitamin D levels to assess overall and *Prakriti* stratified comparison was performed. It was observed that vitamin D deficient subjects in both CAD patients and healthy control groups and more prevalent in controls when compared to the patients. The reason could be the vitamin D supplementation with the standard of care during CAD management in the patients while the healthy controls did not receive any vitamin D supplementation, while earlier studies have reported low vitamin D levels being linked to a higher prevalence and severity of CAD [35]. Further, *Prakriti* stratification of Vitamin D deficient group revealed that *Pitta* CAD patients were at more risk as compare to *Vata* and *Kapha* types (Table 5).

**Table 5:** Vitamin D values retrieved from records and with deficient percentage in overall cases and controls with stratified groups.

Vitamin D ng/mL			Vata		Pitta		*Kapha*	
	Cases	Controls	Cases	Controls	Cases	Controls	Cases	Controls
**N**	65	39	23	10	23	16	29	13
**Mean**	18.84	16.79	17.94	15.6	16.88	19.52	19.73	13.90
**Std. Deviation**	11.18	10.96	10.19	10.30	6.27	13.64	9.13	5.82
**Median**	16.3	15.8	15.65	11.55	15.5	16.2	20.4	14.2
**Quartiles 1**	10.8	8.97	8.97	9.55	12.42	9.2	10.7	8.75
**Quartiles 3**	22.7	19.8	25.22	22.4	20.9	24.4	25.9	18.85
**p-value**	0.633		0.974		0.425		0.047(∗)	
**# p-value compared using independent sample *t*-test (∗) denoted p-value is significant at 5%level of significance**								

Deficiency Index	Total	*Vata*	*Pitta*	*Kapha*
**Cases/Controls**	65/39	23/10	23/16	29/13
**Deficient cases**	58%	59%	73%	37%
**Deficient controls**	76%	70%	61%	90%

Values are expressed as mean ± standard deviation unless otherwise indicated. Median and quartiles represent the 50th, 25th (Q1), and 75th (Q3) percentiles, respectively. Serum vitamin D levels are reported in ng/mL. p-values were calculated using the independent sample *t*-test to compare cases and controls within each *Prakriti* group. A p-value <0.05 was considered statistically significant. Vitamin D deficiency was defined according to standard clinical criteria (serum vitamin D < 20 ng/mL).

#### 3.2.6 Association of genomic results with their biochemical parameters and biomarkers

The association *ADAMTS7, MTHFR* and *VDR* Gene and other parameters like TLC, fasting blood sugar, urea, creatinine, uric acid, HbA1c, MDA, NT-pro BNP were significantly high in CAD patients as compared to controls. However total cholesterol, low density lipoproteins and high-density lipoproteins were significantly high in healthy controls as compared to CAD patients. Overall results demonstrated the connotation of the complex network involved in the disease predisposition in CAD patients and the risks associated with seemingly healthy controls also. Further, *Prakriti* stratification could make more clear inference like association of *VEGFA* gene, *CD40* and *VDR* gene with high IL-6 and increased monocyte lymphocyte ratio demonstrated the association of inflammatory pathways in *Vata Prakriti* CAD patients as risk factors. In *Pitta Prakriti* association of VDR gene and low Vitamin D levels and high alkaline phosphatase depicted vitamin D as a disease predisposing factor in such patients. In *Kapha* association of *MTHFR* gene and disturbed glycemic controls could make an inference of poor glycemic control as disease predisposition factor in *Kapha prakriti* CAD patients (Table 6).

**Table 6:** Association results in overall groups of CAD patients and healthy controls and stratified groups of *Vata, Pitta and Kapha Prakriti* CAD patients and healthy controls.

Group	SNPs	Genes	Biochemical and Biomarkers
**Overall Results**	rs3825807 rs1801133 rs1544410 rs7975232 rs731236	ADAMTS7 gene MTHFR gene VDR gene VDR gene VDR gene	➢ TLC, fasting blood sugar, urea, creatinine, uric Acid, HbA1C, MDA, NT- pro BNP, Monocyte lymphocyte ratio high in CAD patients. ➢ Lymphocyte count, total cholesterol, low density lipoproteins, and high-density lipoproteins were low in CAD patients. ➢ Vitamin D deficiency both in CAD patients and healthy controls.
*Vata Group*	rs1544410 rs3025000 rs1883832 rs7566605	VDR gene VEGFA gene CD40 gene CCDC93; INSIG2 intergenic	➢ Monocyte lymphocyte ratio and IL-6 were hhigh in Vata CAD patients ➢ PCSK9 l and Lppla2 low in Vata CAD patients.
*Pitta Group*	rs1800796 rs7975232 rs86153 rs1544410 rs1883832 rs2066865 rs7521023	IL-6 gene VDR gene XRCC3 gene VDR gene CD40 gene FGG gene CASQ2 gene	➢ Creatinine, alkaline phosphatase, and catalase were high in *Pitta* CAD patients. ➢ Vitamin D was low in *Pitta* CAD patients
*Kapha Group*	rs1801133	MTHFR gene	➢ Fasting blood sugar, uric acid, creatinine, and MDA were high and catalase was low in *Kapha Prakriti* CAD patients. ➢ Vitamin D was low in *Kapha Prakriti* healthy controls.

## 4 Discussion

This study integrates large-scale genomic data mining with Ayurvedic phenotypic stratification to investigate CAD susceptibility. Five polymorphisms namely rs154410, rs7975232, rs731236, rs3825807 and rs1801133 showed significant association with CAD amongst the 255 susceptible SNPs. These five polymorphisms with the enzymes linked to *MTHFR* [36], *ADAMTS7* [37] and *VDR* [35] have been studied in several disease conditions in Indian and other population groups. These genes have been reported to be associated with pathophysiology of CAD but there are only a few studies presenting direct association with CAD in Indian Asian population [38]. Among these five polymorphisms, three SNPs rs1544410, rs7975232 and rs731236 were present at vitamin D receptor (*VDR*) gene of chromosome 12. rs1544410, also known as the *Bsm*I polymorphism, with AA, AG and GG genotypes has been reported to be associated with increased, intermediate, and low risk of low bone mineral density disorders. rs7975232 is also called as *Apa*I polymorphism and rs731236 is known as Taq1 [39]. Studies of combined associations of these SNPs with various pathological conditions have been presented in different diseases like in female infertility [40], multiple myeloma [41], pulmonary tuberculosis [42], diabetes [43], psoriasis [44] and hepatocellular carcinoma [45].

The polymorphism rs1801133 is also known as C677T, Ala222Val, or A222V. It is a well-studied and relatively common variant in the *MTHFR* gene which is crucial for folate metabolism [46]. There is a long list of disorders in various populations across the world linking rs1801133. The reduced activity of this SNP has been associated with the conditions like autism, cancer, cleft lip, cleft palate, CAD, dementia, depression, and migraine [47]. It is a thermolabile variant and each alternate allele of this polymorphism has been observed to be associated with increased serum homocysteine levels [48], which is linked to an elevated risk of CAD. The *MTHFR* gene transcript codes for the enzyme called methylenetetrahydrofolate reductase or *MTHFR*. This enzyme is responsible for conversion of the co-substrate that helps in the re-methylation of homocysteine back to methionine [49]. It has been reported that heterozygous genotype of this polymorphism codes for around 35% of reduced enzyme activity and the homozygous genotype for alternate allele codes for around 70% reduced enzyme activity leading to hyper-homocysteinemia. In the present set of our studied population, the rs1801133 polymorphism was expressed with AA, AG and GG genotypes, and was observed to be protective. Although the alternate allele frequency was around 15% in CAD patients and 29% in healthy controls, there were no homozygous for alternate allele in the healthy controls. However, 4% of the alternate allele were homozygous genotype in the CAD patients [50].

Higher homocysteine levels been reported are associated with increased risk and severity of CAD [51]. The role of homocysteine levels in endothelial dysfunction has also been established in literature thus providing evidence towards its role in CAD [[52], [53], [54]]. In addition to the previously reported associations with CAD, we also tried to check the clinical significance of this SNP in hyper-homocysteinemia [55]. With a SIFT score [56] of 0.02 and a CADD score [57] of 27.3, this polymorphism was found to be detrimental. It was also predicted to have a negative effect with a PolyPhen score [58] of 0.943. As per the conventional guidelines of American College of Medical Genetics and Genomics (ACMG) [59] this polymorphism is not likely a pathogenic variant because of allelic frequency >5%. However, as per the expanded ACMG variant classification guidelines that have been used for common variants, this can be classified as a likely predisposing genetic variation for increased levels of homocysteine, and hence, for CAD.

Regarding rs3825807 present at *ADAMTS7* gene of chromosome 15, the results of the present study had demonstrated a positive expression of this polymorphism is indicating a protective role. Even being in the list of susceptible SNPs, some where our findings agree with the results of an earlier study showing an association of rs3825807 of *ADAMTS7* with thrombospondin-5 cleavage, vascular smooth muscle cell migration and ultimately decreasing the risk for CAD [60]. It is important to note that this was a cross-sectional study aimed at identifying population-specific associations between known susceptible SNPs and CAD in an Indian cohort. While we observed a significant association of the rs3825807 polymorphism in the *ADAMTS7* gene, our design does not allow us to infer causality or temporal risk. Previous longitudinal studies, such as that by Iwanicka et al., have demonstrated the prognostic relevance of *ADAMTS7* variants in predicting CAD over extended follow-up periods [61,62]. Therefore, our findings should be considered preliminary and hypothesis-generating. Our results underscore the need for future prospective follow-up studies with larger cohorts to determine the predictive and prognostic significance of these SNPs in CAD development and progression within Indian and other populations.

IL-6 was significantly higher only in *Vata* CAD patients. IL-6 has also been shown as a predictor of increased oxidative stress. while IL-6 levels were high, indicating an involvement of the immune and inflammatory pathways for disease predisposition in *Vata* patients. Previous studies have also given lead towards getting early readouts of disease predisposition even in healthy individuals when they were phenotypically *Prakriti* stratified. Additionally, a research study provided validation for the significance of *Prakriti* based stratification in evaluating the distinct responses of autonomic cardiac modulation in different postures among healthy individuals [63,64]. Creatinine and uric acid were increased in CAD patients and *Prakriti* stratification showed increased levels in *Vata* CAD patients which are associated with *CD40* gene. PCSK9 and LpPLA2 were comparatively low in Vata patients. Further, as per biochemical results observations, increased IL-6 and GSA results demonstrated association of rs3025000 of VEGFA gene and there is coherence between IL-6 and rs3025000 of *VEGFA* gene.

Studies have evident the association of rs1800796 of *IL-6* gene and rs1883832 polymorphism of *CD 40* gene with higher values of creatine and a major risk in coronary as well renal health [65]. Studies show inverse association of alkaline phosphatase and Vitamin D deficiency [66]. Vitamin D deficient ratio was observed in *Pitta* CAD patients as compared to controls. Moreover, alkaline phosphatase was higher in *Pitta* CAD group, a symptom of vitamin D deficiency rather than in *Vata* and *Kapha* groups. In GSA results, association of different polymorphisms of *VDR* gene have justified interconnection of biochemical and genetic variants. Further, fasting blood sugar and HbA1c was higher in all CAD patients. However, the fasting blood sugar levels were higher in patients with *Kapha* predominant as compare to other cases. MDA was higher in CAD patients compared to healthy controls and *Prakriti* segregation showed significantly higher levels only in *Kapha* patients. An increase in level of free radicals due to oxidative stress causes increased production of MDA. PCSK9 levels and catalase were also higher in *Kapha* patients. Recent evidence showed regulatory effects of PCSK9 on redox system which may be the predisposing factor among *Kapha* individuals. Further, all these features are in coherence with the association with *MTHFR* gene.

Further, we analyzed only the associated genotypes of rs731236 polymorphism in the VDR gene, which may be expressed differently in CAD patients and healthy controls. While on *Prakriti* stratification gave more insights as there were higher Serum alkaline phosphatase and low Vitamin D in *Pitta Prakriti* group of CAD patients which may be predisposing risk factor for CAD in *Pitta Prakriti* individuals. Further, *Kapha* controls were having high percentage of deficient individuals and poor glycemic control both may be alarming signs for seemingly heathy individuals because studies evident the allelic variation in VDR gene in diabetics are associated with increased risk for CAD [67]. These hypotheses highlight the potential for integrating genomic biomarkers with Ayurvedic constitutional assessment to develop personalized preventive strategies for CAD, although further longitudinal and interventional studies are required to validate these approaches.

This study has certain limitations, including a relatively small sample size, a cross-sectional design, and recruitment of participants from a single center. The results may vary if participants from different ethnic backgrounds are stratified. In the present study, the sample size was small, and being a tertiary care hospital the participants originated from diverse native regions. Therefore, conducting subgroup analyses based on ethnicity would be challenging and could lead to overinterpretation of limited data. Therefore, large multicenter studies integrating genomics and *Prakriti* phenotyping are needed to validate these findings.

## 5 Conclusion

Elucidation of the susceptibility in terms of genetic variants in CAD is very difficult due to the involvement of a wide genetic area and several SNPs. The present study was targeted to identify susceptible polymorphisms associated with CAD. A comprehensive analysis identified 255 SNPs across 134 genes associated with CAD in diverse populations. In the Indian context, *VDR, MTHFR, and ADAMTS7* genes have been highlighted as significant risk factors for CAD while these genetic variations have been linked to other diseases within the Indian population, their specific role in CAD remains underexplored. Therefore, conducting extensive studies focusing on these variants could provide deeper insights into their contribution to CAD susceptibility in population specific manner. Several research works have found distinct genetic and metabolic profiles among healthy individuals grouped by *Prakriti*. In the present study, the *Prakriti* stratification gave more elaborated results in genomics and which were very much comparable with their biochemical parameters and biomarkers both for CAD patients as well for healthy controls. Therefore, this phenotypic screening tool can be used as an important biomarker in risk prediction and to assess the disease predisposition in CAD patients and in seemingly healthy individuals. Large scale studies in multiple settings are proposed to be planned for further validation of the inferences drawn from the present study.

## Ethics approval and consent to participate

Ethical approval was obtained from the Institute Ethics Committee, All India Institute of Medical Sciences, New Delhi, India (IEC-510/05.10.2018, RP-40/2018). Signed written informed consent was obtained from all the participants of the study. All procedures performed in this study were in accordance with the ethical standards of the institutional ethics committee and with the 1964 Helsinki Declaration and its later amendments or comparable ethical standards.

## Author contributions

Pamila Dua conceptualized the study, did manual literature search, design, conduct, data procurement, data analysis, interpretation, drafting of the manuscript. Deepshikha Punera helped in GSA data analysis and annotation. Dhwani Dholakia did the bioinformatic part of the literature search. Archana Vats assisted in GSA. Shivam Pandey did the statistical analyses. Sandeep Seth helped to design of the study and revision of the manuscript. Bhavana Prasher contributed to design the study helped in data acquisition, data analysis and revising the manuscript. Mitali Mukerji contributed to design and data acquisition. SK Maulik critically reviewed and revised the manuscript. KH Reeta contributed to design, analysis, interpretation, and critical revision of the manuscript. All authors reviewed and approved the final manuscript and agree to be accountable for all aspects of the work.

## Clinical trial registry -India (CTRI/2019/01/016866)

Institute ethics committee, AIIMS, New DELHI (IEC510/05/10, 2018, RP-40/2018)

## Declaration of generative AI in scientific writing

The authors declare that this article did not use generative AI or AI- assisted technologies.

## Funding sources

Dr Pamila Dua received Women Scientist-A fellowship (LS 376/2018) from Department of Science and Technology, Government of India for this research work. Dr. Deep Shikha Punera received fellowship (COE 0183) from, Ministry of AYUSH, Government of India.

## Appendix A Supplementary data

**Supplementary file 1**: Detailed data of the polymorphisms retrieved from literature search. Multimedia component. 1

**Supplementary file 2**: List of 255 susceptible polymorphisms with associated genes. Multimedia component. 2

**Supplementary file 3**: Details of the data for calculation of cumulative Odds Ratio. Multimedia component. 3

**Supplementary file 4**: Details of 5 polymorphisms amongst 255 SNPs with significant association in the study population with details of 16 polymorphisms with p < 0.00001 in our population set and global allele frequencies of different populations from the 1000 Genomes Project and gnom AD database. [GC: Genome Correction; BON F: Bonferroni test; Sidak: Dunn-sidak-test; FDR: False Discovery Rate; AFR: African/African American; AMR: Admixed American; EAS: East Asians; SAS: South Asian; EUR: European; FIN: Finnish; NFE: Non-Finnish European; OTH: Other (Population not assigned)]. Multimedia component. 4
