# Supplementary material for: Association of coronary artery disease related single nucleotide-polymorphisms with extreme Prakriti types: Insights from a case control study
Source: J Ayurveda Integr Med. 2026 Jul 8;17(4):101371. doi: 10.1016/j.jaim.2026.101371 (PMC13356639; doi:10.1016/j.jaim.2026.101371)
Supplement: Supplementary file 1 — Detailed data of the polymorphisms retrieved from literature search. Multimedia component. 1 [file mmc1.pdf]

Supplementary File 1

	SNPs from literature search with OR>1							Participants				
S.No.	PMID	doi	Reference	SNPs	Chromosome	Functional Reference	Gene Reference	Cases (n)	Controls (n)	OR	Population/Place	Continent
1	17259397	https://doi.org/10.2337/db06-0946	1	rs5029930	6	intronic	TNFAIP3	240	239	2.3	Boston	North America, Europe
				rs610604	6	intronic	TNFAIP3	240	239	2.4	Boston	North America, Europe
				rs583522	6	intronic	TNFAIP3	332	239	1.7	Boston	North America, Europe
				rs610604	7	intronic	TNFAIP3	332	239	1.1	Italy	North America, Europe
				rs5029930	6	intronic	TNFAIP3	332	239	1.4	Italy	North America, Europe
				r5029933	6	intronic	TNFAIP3	332	239	1.8	Boston	North America, Europe
2	17357071	http://dx.doi.org/10.1086/512981	2	rs9289231	3	intergenic	ROPN1; KALRN	301	546	3.1	North Carolina	North America
				rs6810298	3	intronic	ROPN1	301	546	1.7	North Carolina	North America
				rs17376453	3	intronic	ROPN1	301	546	2.1	North Carolina	North America
		*rs4499545 is the current name for SNP rs7434266		rs7434266*	3	intronic	ROPN1	301	546	1.8	North Carolina	North America
				rs7613868	3	intergenic	ROPN1; KALRN	301	546	2.6	North Carolina	North America
				rs12634530	3	intergenic	ROPN1; KALRN	301	546	1.3	North Carolina	North America
				rs12637456	3	intergenic	ROPN1; KALRN	301	546	2.2	North Carolina	North America
				rs13075202	3	intergenic	KALRN	301	546	2.1	North Carolina	North America
				rs1444768	3	intronic	KALRN	301	546	1.8	North Carolina	North America
				rs1444754	3	intronic	KALRN	301	546	1.7	North Carolina	North America
				rs4234218	3	intronic	KALRN	301	546	1.4	North Carolina	North America
3	17634449	https://doi.org/10.1056/nejmoa072366	3	rs599839	1	downstream	PSRC1	2807	4582	1.13	Europe	Europe
				rs3008621	15	intronic	SMAD3	2807	4582	1.1	Europe	Europe
				rs2943634	1	intronic	MIA3	2807	4582	1.2	Europe	Europe
				rs501120	10	intergenic	LINC00841; C10orf142	2807	4582	1.11	Europe	Europe
				rs6922269	6	intronic	MTHFD1L	2807	4582	1.23	Europe	Europe
				rs1333049	9	intergenic	CDKN2B-AS1; DMRTA1	2807	4582	1.37	Europe	Europe
4	18264662	https://doi.org/10.1007/s10038-008-0248-4	4	rs1333049	9	intergenic	CDKN2B-AS1; DMRTA1	604	1151	1.33	Japan	Asia
				rs1333049	9	RXRA	CDKN2B-AS1	679	706	1.44	Korea	Asia
5	18318662	https://doi.org/10.1042/cs20070468	5	rs17222814	12	intronic	LTA4H	1211	1015	1.5	Europe	Europe
				rs10507391	13	intronic	ALOX5AP	1211	1015	1.2	Europe	Europe
				rs4769874	13	intronic	ALOX5AP	1211	1015	1.23	Europe	Europe
				rs9551963	13	intronic	ALOX5AP	1211	1015	1.22	Europe	Europe
				rs17216473	13	intronic	ALOX5AP	1211	1015	1.35	Europe	Europe
				rs9315050	13	intronic	ALOX5AP	1211	1015	1.05	Europe	Europe
6	18369664	https://doi.org/10.1007/s00439-008-0489-5	6	rs12762303	10	upstream	ALOX5	1552	1583	1.32	White	Europe
				rs12762303	10	upstream	ALOX5	1552	1583	1.46	Hispanic	Europe
				rs12762303	10	upstream	ALOX5	1552	1583	1.29	Mixed	Europe
				rs12762303	10	upstream	ALOX5	1552	1583	1.22	Mixed Other	Europe
				rs12762303	10	upstream	ALOX5	1552	1583	1.19	Combined	Europe
				rs2228064	10	exonic	ALOX5	1552	1583	1.24	Blacks	Europe
				rs41526545	10	intronic	ALOX5	1552	1583	1.1	Whites	Europe
				rs41526545	10	intronic	ALOX5	1552	1583	1.27	Mixed	Europe
				rs41526545	10	intronic	ALOX5	1552	1583	1.56	Mixed Other	Europe

				rs2029253	10	intronic	ALOX5	1552	1583	1.35	Hispanic	Europe
				rs2029253	10	intronic	ALOX5	1552	1583	1.84	Asians	Europe
				rs2029253	10	intronic	ALOX5	1552	1583	1.31	Mixed	Europe
				rs2029253	10	intronic	ALOX5	1552	1583	1.03	Combined	Europe
				rs28395866	10	intronic	ALOX5	1552	1583	1.27	Whites	Europe
				rs28395866	10	intronic	ALOX5	1552	1583	1.21	Blacks	Europe
				rs28395866	10	intronic	ALOX5	1552	1583	1.04	Hispanic	Europe
				rs28395866	10	intronic	ALOX5	1552	1583	1.03	Mixed	Europe
				rs2229136	10	exonic	ALOX5	1552	1583	1.12	Whites	Europe
				rs2229136	10	exonic	ALOX5	1552	1583	1.32	Hispanics	Europe
				rs2229136	10	exonic	ALOX5	1552	1583	1.03	Combined	Europe
				rs4769055	13	exonic	ALOX55AP	1552	1583	1.34	Blacks	Europe
				rs4769055	13	exonic	ALOX55AP	1552	1583	1.18	Hispanics	Europe
				rs10507391	13	exonic	ALOX55AP	1552	1583	1.17	Blacks	Europe
				rs10507391	13	exonic	ALOX55AP	1552	1583	1.03	Mixed Hispanic	Europe
				rs3803277	13	intronic	ALOX55AP	1552	1583	1.38	Mixed Hispanic	Europe
				rs3803277	13	intronic	ALOX55AP	1552	1583	1.13	Blacks	Europe
				rs3803277	13	intronic	ALOX55AP	1552	1583	1.03	Combined	Europe
				rs3803278	13	intronic	ALOX55AP	1552	1583	1.05	Whites	Europe
				rs3803278	13	intronic	ALOX55AP	1552	1583	1.38	Mixed Hispanics	Europe
				rs3803278	13	intronic	ALOX55AP	1552	1583	1.07	Asians	Europe
				rs3803278	13	intronic	ALOX55AP	1552	1583	1.04	Combined	Europe
				rs12721458	13	intronic	ALOX55AP	1552	1583	1.14	Mixed	Europe
				rs1132340	13	3'-UTR	ALOX5	1552	1583	1.48	Hispanics	Europe
7	18449426	https://doi.org/10.1160/th07-11-0686	7	rs7802307	7	ncRNA_intronic	LOC541472	284	40	3.676	India	Asia
8	18459066	https://doi.org/10.1007/s11033-008-9259-7	8	rs2383207	9	ncRNA_intronic	CDKN2B-AS1	212	232	1.631	China	Asia
				rs10757278	9	intergenic	CDKN2B-AS1; DMRTA1	212	232	1.955	China	Asia
9	18505420	https://doi.org/10.1111/j.1469-1809.2008.00454.x	9	rs2383206	9	ncRNA_intronic	CDKN2B-AS1	310	560	1.67	America	North America
				rs2383207	9	ncRNA_intronic	CDKN2B-AS1	310	560	1.72	America	North America
				rs10757274	9	ncRNA_intronic	CDKN2B-AS1	310	560	1.78	America	North America
				rs10757278	9	intergenic	CDKN2B-AS1; DMRTA1	310	560	1.76	America	North America
10	18612189	https://doi.org/10.1536/ihj.49.313	10	rs10761600	10	intronic	ARID5B	475	310	2.2	Japan	Asia
				rs7087507	10	intronic	ARID5B	475	310	1.82	Japan	Asia
11	18637188	https://doi.org/10.1186/1471-2261-8-15	11	rs4343	17	exonic	ACE	298	510	1.56	Madeira Island, Portugal	Africa, Europe
				rs4340	17	intronic	ACE	298	510	1.795	Madeira Island, Portugal	Africa, Europe
12	18652946	https://doi.org/10.1016/j.jacc.2007.11.087	12	rs1333049	9	intergenic	CDKN2B-AS1; DMRTA1	103	666	1.87	Austria	Europe
13	18654002	https://doi.org/10.1253/circj.72.1213	13	rs17465637	1	intronic	MIA3	2326	10427	1.45	Japan	Asia
				rs1333049	9	intergenic	CDKN2B-AS1; DMRTA1	2326	10427	1.47	Japan	Asia
				rs501120	10	intergenic	LINC00841; C10orf142	2326	10427	1.22	Japan	Asia
14	19033013	https://doi.org/10.1016/j.ahj.2008.07.006	14	rs2383206	9	ncRNA-intronic	CDKN2B-AS1	1011	545	1.69	Utah, America	North America
				rs2383206	9	ncRNA-intronic	CDKN2B-AS2	1011	545	1.99	Utah, America	North America
15	19033589	https://doi.org/10.1001/jama.2008.649	15	rs2383206	9	ncRNA-intronic	CDKN2B-AS1	322	412	1.39	Israel	Asia
16	19048183	https://doi.org/10.1007/s10238-008-0022-0	16	rs5498	19	exonic	ICAM1	191	203	4.8	Poland	Europe
17	19475673	https://doi.org/10.1002/ana.21590	17	rs1537378	9	ncRNA-intronic	CDKN2B-AS1	4318	4305	1.11	Europe and North America	Europe, North America
18	19506371	https://doi.org/10.1159/000223437	18	rs1799864	3	exonic	CCR2	77	248	6.14	African Americans	Africa, America
				rs2857656	17	upstream	CCL2	77	248	2.09	African Americans	Africa, America

19	19548844	https://doi.org/10.1515/cclm.2009.215	19	rs1333049	9	intergenic	CDKN2B-AS1; DMRTA1	520	560	1.48	China	Asia
20	19563249	https://doi.org/10.1089/dna.2009.0908	20	rs2228570	12	exonic	VDR	152	212	1.23	China	Asia
21	19571538	https://doi.org/10.1159/000226610	21	rs3812316	7	exonic	MLXIPL	352	152	2.96	China	Asia
22	19575309	https://doi.org/10.1007/s11033-009-9597-0	22	rs2283228	11	intronic	KCNQ1	66	90	4.569	China	Asia
				rs2237897	11	intronic	KCNQ1	66	90	1.569	China	Asia
				rs2237895	11	intronic	KCNQ1	66	90	1.25	China	Asia
23	19624571	https://doi.org/10.1111/j.1469-1809.2009.00534.x	23	rs1048990	14	5'-UTR	PSMA6	1071	929	1.18	Saudi Arabia	Asia
				rs7157492	14	intronic	KIAA0391	1071	929	1.52	Saudi Arabia	Asia
				rs4981283	14	intronic	KIAA0391	1071	929	1.08	Saudi Arabia	Asia
24	19687159	https://doi.org/10.1093/eurheartj/ehp316	24	rs187238	11	upstream	IL18	663autopsies		3.08	Finland	Europe
25	19713974	https://doi.org/10.1038/jhg.2009.87	25	rs599839	1	downstream	PSRC1	551	1384	1.28	Japan	Asia
				rs599839	1	downstream	PSRC1	1255	2058	1.09	Both Japan and Korea	Asia
				rs17465637	1	intronic	MIA4	760	641	1.09	Korea	Asia
				rs17465637	2	intronic	MIA5	760	641	1.09	Japan	Asia
				rs17465637	3	intronic	MIA6	760	641	1.09	Both Japan and Korea	Asia
				rs17672135	1	intronic	FMN2	740	675	1.11	Korea	Asia
				rs2943634	2	noncoding	AC068138.1 pseudogene	553	1373	1.12	Japan	Asia
				rs2943634	2	noncoding	AC068138.1 pseudogene	1222	2009	1.05	Both Japan and Korea	Asia
				rs383830	5	intergenic	FAM174A	596	1392	1.09	Japan	Asia
				rs501120	10	intergenic	LINC00841; C10orf142	565	1387	1.09	Japan	Asia
				rs501120	11	intergenic	LINC00841; C10orf143	565	1387	1.11	Korea	Asia
				rs501120	12	intergenic	LINC00841; C10orf144	565	1387	1.09	Both Japan and Korea	Asia
				rs8055236	16	intronic	CDH13	620	1352	1.04	Japan	Asia
				rs11066001	12	intronic	BRAP	603	1402	1.65	Japan	Asia
				rs11066001	12	intronic	BRAP	757	713	1.68	Korean	Asia
				rs11066001	12	intronic	BRAP	1360	2115	1.63	Both Japan and Korea	Asia
26	19782985	https://doi.org/10.1016/j.atherosclerosis.2009.09.005	26	rs34851361	15	exonic	MEF2A	1186	885	1.22	Saudi Arabia	Asia
				rs1059759	15	3'-UTR	MEF2A	1186	885	1.21	Saudi Arabia	Asia
27	19786296	https://doi.org/10.1016/j.thromres.2009.09.002	27	rs1613662	9	exonic	PTGS1	105	132	1.88	Germany	Europe
				rs 6025	3	exonic	P2RY12	105	132	1.718	Germany	Europe
				rs1799963	5	exonic	ITGA2	105	132	1.019	Germany	Europe
				rs6785930	3	synonymous	P2RY12	105	132	1.005	Germany	Europe
				rs1126643	5	synonymous	ITGA2	105	132	1.169	Germany	Europe
28	19901189	https://doi.org/10.1161/circulationaha.109.879049	28	rs10757274	9	intronic	CDKN2B-AS1	516	1540	1.52	USA	North America
29	19924244	https://doi.org/10.1371/journal.pone.0007697	29	rs7903146	10	intronic	TCF7L2	889	559	1.325	Brazil	South America
30	19954776	https://doi.org/10.1016/j.atherosclerosis.2009.11.004	30	rs3744700	17	intronic	CXCL16	1176	850	1.77	China	Asia
				rs2304973	17	intergenic	CXCL16, ZMYND15	1176	850	1.02	China	Asia
31	19959123	https://doi.org/10.1016/j.hrthm.2009.08.031	31	rs9838682	3	intergenic	RBMS3; LINC01985	89	520	1.66	San Francisco	North America
32	20032323	https://doi.org/10.1056/nejmoa0902604	32	rs10455872	6	intronic	LPA	3145	3352	1.7	Finland	Europe
				rs3798220	6	exonic	LPA	3145	3352	1.92	Finland	Europe
33	20109173	https://doi.org/10.1186/1471-2350-11-15	33	rs11274804	2	intronic	AC007254.3	257	413	2.02	Estonia	Europe
34	20167083	https://doi.org/10.1186/1471-2350-11-28	34	rs2070006	4	intergenic	FGA; FGG	305	305	1.51	Greece	Europe
35	20417517	https://doi.org/10.1016/j.atherosclerosis.2010.03.030	35	rs671	12	exonic	ALDH2	417	448	1.85	China	Asia
36	20466371	https://doi.org/10.1016/j.atherosclerosis.2010.04.004	36	rs6507931	18	intronic	LIPG	1138	2237	1.09	China	Asia
37	20576952	https://doi.org/10.1161/strokeaha.110.583096	37	rs11206510	1	intergenic	BSND; PCSK9	1543	1240	1.49	China	Asia

38	20592455	https://doi.org/10.3233/dma-2010-0710	38	rs11887534	2	exonic	ABCG8	213	220	2.44	India	Asia
39	20676957	https://doi.org/10.1007/s00380-009-1223-5	39	rs7217186	17	intronic	ALOX15	519	608	2.6	China	Asia
				rs2619112	17	intronic	ALOX15	519	608	1.2	China	Asia
40	20691078	https://doi.org/10.1186/1475-2840-9-33	40	rs1333049	9	intergenic	CDKN2B-AS1; DMRTA1	1616	430	1.524	China	Asia
41	20718794	https://doi.org/10.1111/j.1399-0004.2010.01509.x	41	rs1994016	15	intronic	ADAMTS7	311	443	1.14	India	Asia
42	20729229	https://doi.org/10.1093/eurheartj/ehq272	42	rs10757278	9	intergenic	CDKN2B-AS1; DMRTA1	1830	504	1.17	Caucasians	USA, Europe
43	20810930	https://doi.org/10.1182/blood-2010-03-277079	43	rs688	19	exonic	LDLR	692	291	1.67	Italy	Europe
44	20858033	https://doi.org/10.1089/dna.2010.1046	44	rs10757278	9	intergenic	CDKN2B-AS1; DMRTA1	414	408	2.2	India	Asia
45	20951690	https://doi.org/10.1016/j.cca.2010.10.008	45	rs3767443	1	intronic	CREG1	1161	960	1.27	China	Asia
				rs3753921	1	intronic	CREG1	1161	960	1.51	China	Asia
46	21091218	https://doi.org/10.1089/dna.2010.1129	46	rs1883832	20	5'-UTR	CD40	160	180	2.779	China	Asia
				rs1883832	20	5'-UTR	CD41	160	180	1.554	China	Asia
47	21252575	https://doi.org/10.1159/000321206	47	rs1042522	17	exonic	TP53	209	216	2	South America	South America
48	21264445	https://doi.org/10.1160/th10-10-0641	48	rs17465637	1	intronic	MIA3	3657	1211	1.12	Germany	Europe
49	21270277	https://doi.org/10.2337/db10-0185	49	rs10757283	9	intergenic	CDKN2B-AS1; DMRTA1	1093	1695	1.18	China	Asia
				rs10811661	9	intergenic	CDKN2B-AS1; DMRTA1	1093	1695	1.19	China	Asia
				rs2383208	9	intergenic	CDKN2B-AS1	1093	1695	1.11	China	Asia
50	21273694	https://doi.org/10.1007/s12041-010-0062-6	50	rs11568828	17	upstream	GH1	299	231	3.13	India	Asia
				rs6171	17	5'-UTR	GH1	299	231	3.13	India	Asia
				rs2005172	17	upstream	GH1	299	231	3.13	India	Asia
				rs2005171	17	upstream	GH1	299	231	3.13	India	Asia
51	23091611	https://doi.org/10.1371/journal.pone.0047193	51	rs10757274	9	ncRNA-intronic	CDKN2B-AS1	95	110	1.8	China	Asia
52	23123830	https://doi.org/10.1016/j.cca.2012.10.042	52	rs2236242	14	intronic	SERPINA12	360	460	1.32	China	Asia
				rs2236242	14	intronic	SERPINA13	360	460	1.43	China	Asia
53	23195133	https://doi.org/10.1016/j.clinbiochem.2012.11.009	53	rs8034928	15	intronic	IL16	651	428	1.47	China	Asia
54	23337689	https://doi.org/10.1016/j.clinbiochem.2013.01.004	54	rs6903956	6	intronic	ADTRP	122	118	2.03	China	Asia
55	23352876	https://doi.org/10.1016/j.diabet.2012.11.004	55	rs731236	12	exonic	VDR	220	483	1.75	Paris	Europe
				rs7975232	12	intronic	VDR	220	483	1.47	Paris	Europe
				rs1544410	12	intronic	VDR	220	483	1.4	Paris	Europe
56	23383292	https://doi.org/10.1371/journal.pone.0055869	56	rs174460	11	intronic	FADS3	505	510	1.896	China	Asia
57	23554780	https://doi.org/10.7555/jbr.26.20110116	57	rs13306541	8	5'-UTR	MSR1	402	400	1.97	China	Asia
				rs416748	8	upstream	MSR1	402	400	1.8	China	Asia
58	23590551	https://doi.org/10.1186/1475-2840-12-67	58	rs266729	3	upstream	ADIPOQ	560	550	1.64	China	Asia
				rs182052	3	intronic	ADIPOQ	560	550	1.18	China	Asia
				rs12495941	3	intronic	ADIPOQ	560	550	1.04	China	Asia
				rs1063539	3	3'-UTR	ADIPOQ	560	550	1.06	China	Asia
				rs3821799	3	intronic	ADIPOQ-AS1	560	550	1.07	China	Asia
59	23593153	https://doi.org/10.1371/journal.pone.0059905	59	rs6730157	2	intronic	RAB3GAP1	948	3050	1.6	Portland	North America
				rs2077316	10	intronic	ZNF365	948	3050	2.41	Portland	North America
60	23611467	https://doi.org/10.1042/cs20120572	60	rs2043211	19	exonic	CARD8	202	198	1.2	Sweden	Europe
61	23625196	https://doi.org/10.1007/s11010-013-1665-z	61	rs662	7	exonic	PON1	121	108	3.2	Saudi Arabia	Asia
62	23787071	https://doi.org/10.1186/1475-2840-12-93	62	rs1333049	9	intergenic	CDKN2B-AS1; DMRTA1	135	73	8.16	Thailand	Asia
63	23819814	https://doi.org/10.1089/gtmb.2013.0109	63	rs3918242	20	intergenic	ZNF335; MMP9	258	100	2.005	China	Asia
64	23880405	https://doi.org/10.1016/j.clinbiochem.2013.07.011	64	rs10903323	8	intronic	MSRA	435	480	1.31	China	Asia
				rs699947	6	intergenic	MRPS18A; VEGFA	435	480	1.26	China	Asia
				rs3025039	6	3'-UTR	VEGFA	435	480	1.02	China	Asia
65	23881440	https://doi.org/10.1177/0300060513483405	65	rs8034928	15	intronic	IL16	326	341	2	China	Asia
				rs11556218	15	exonic	IL16	326	341	2.41	China	Asia

66	23968400	https://doi.org/10.1186/1475-2840-12-119	66	rs9770242	7	upstream	NAMPT	406	188	1.46	Brazil	South America
				rs9770242	7	upstream	NAMPT	424	434	1.02	North America	North America
67	24040261	https://doi.org/10.1371/journal.pone.0074498	67	rs5744292	11	3'-UTR	IL18	1001	0	1.87	Norway	Europe
				rs3918242	20	intergenic	ZNF335; MMP9	1001	0	1.87	Norway	Europe
68	24069331	https://doi.org/10.1371/journal.pone.0074703	68	rs2246833	10	intronic	LIPA	899	667	1.41	Caucasians and Asians	Asia, America
				rs1412444	10	intronic	LIPA	899	667	1.6	Caucasians and Asians	Asia, America
69	24135527	https://doi.org/10.1159/000352054	69	rs7961581	12	intergenic	TSPAN8; LGR5	755	no controls	1.26	Chicago	North America
				rs11206510	1	intergenic	BSND; PCSK9	755	no controls	1.28	Chicago	North America
				rs2075650	10	intergenic	CDC123; CAMK1D	755	no controls	1.14	Chicago	North America
				rs12740374	1	3'-UTR	CELSR2	755	no controls	1.11	Chicago	North America
				rs12779790	10	intergenic	LINC00841; C10orf142	755	no controls	1.03	Chicago	North America
				rs3846663	5	3'-UTR	HMGCR	755	no controls	1.08	Chicago	North America
				rs12779790	10	intergenic	CDC123; CAMK1D	755	no controls	1.04	Chicago	North America
				rs1470579	3	intronic	IGF2BP2	755	no controls	1.04	Chicago	North America
				rs1864163	16	intronic	CETP	755	no controls	1.16	Chicago	North America
				rs2383207	9	ncRNA-intronic	CDKN2B-AS1	755	no controls	1.11	Chicago	North America
				rs1883025	9	intronic	ABCA1	755	no controls	1.04	Chicago	North America
70	24151447	https://doi.org/10.7150/ijms.7044	70	rs5888	12	exonic	SCARB1	601	582	1.76	China	Asia
71	24155913	https://doi.org/10.1371/journal.pone.0076966	71	rs2070600	6	exonic	AGER	1142	1106	1.26	China	Asia
				rs184003	6	intronic	AGER	1142	1106	1.59	China	Asia
				rs1800625	6	upstream-downstream	AGER; PBX2	1142	1106	1.75	China	Asia
				rs1800624	6	upstream-downstream	AGER; PBX2	1142	1106	1.06	China	Asia
72	24156251	https://doi.org/10.1186/1476-511X-12-153	72	rs565470	13	intronic	COL4A1	471	624	1.993	China	Asia
				rs605143	13	intronic	COL4A1	471	624	1.369	China	Asia
73	24201118	https://doi.org/10.1097/mca.0000000000000056	73	rs4994	8	exonic	ADRB3	1978	642	1.7	India	Asia
				rs1801253	10	missense	ADRB1	2108	691	1.8	India	Asia
				rs1800544	10	exonic	ADRA2	2108	691	1.1	India	Asia
74	24281401	https://doi.org/10.1177/1479164113514232	74	rs11212617	11	intronic	C11orf65	242	no controls	2.7	Germany	Europe
75	24283500	https://doi.org/10.1186/1476-511x-12-176	75	rs1864163	16	intronic	CETP	558	557	1.16	China	Asia
				rs1800775	16	upstream	CETP	732	776	1.183	China	Asia
				rs5882	16	missense	CETP	707	706	1.01	China	Asia
76	24315498	https://doi.org/10.1016/j.thromres.2013.11.017	76	rs25487	19	exonic	XRCC1	1142	1106	1.31	China	Asia
				rs1801133	1	exonic	MTHFR	1142	1106	1.54	China	Asia
				rs4846049	1	3'-UTR	MTHFR	1142	1106	1.94	China	Asia
				rs861539	14	exonic	XRCC3	1142	1106	1.07	China	Asia
				rs1799782	19	exonic	XRCC1	1142	1106	1.53	China	Asia
				rs25487	19	missense	XRCC1	1142	1106	1.11	China	Asia
77	24327156	https://doi.org/10.3760/cma.j.issn.1003-9406.2013.06.020	77	rs1800872	1	upstream	IL10	294	172	2.45	China	Asia
78	24353682	https://doi.org/10.12669/pjms.294.3650	78	rs3848180	15	intronic	IL16	260	281	1.79	China	Asia
				rs8034928	15	intronic	IL16	260	281	1.87	China	Asia
79	24361877	https://doi.org/10.1016/j.bbrc.2013.12.065	79	rs1801157	10	3'-UTR	CXCL12	592	625	2.28	China	Asia
80	24395629	https://doi.org/10.1002/dmrr.2520	80	rs3805486	5	intronic	PRKAA1	260	144	1.16	China	Asia
				rs13361707	5	intronic	PRKAA1	260	144	1.17	China	Asia
81	24444446	https://doi.org/10.1016/j.hrthm.2014.01.015	81	rs7521023	1	3'-UTR	CASQ2	114	311	2.7	San Francisco	North America
				rs6665970	1	intronic	CASQ3	114	311	1.4	San Francisco	North America
				rs9428090	1	intronic	CASQ4	114	311	2.7	San Francisco	North America
82	24447667	https://doi.org/10.1016/j.mrfmmm.2014.01.001	82	rs2910164	5	intronic	MIR146A	295	283	1.39	China	Asia
				rs11614913	12	ncRNA-intronic	MIR196A2	295	283	1.05	China	Asia

83	24475106	https://doi.org/10.1371/journal.pone.0086332	83	rs1122608	19	intronic	SMARCA4	2365	2678	1.02	China	Asia
				rs12190287	6	3'-UTR	TCF21	2365	2678	1.19	China	Asia
				rs12413409	10	intronic	CNNM2	2365	2678	1.12	China	Asia
				rs4977574	9	intronic	CDKN2B-AS1	2365	2678	1.5	China	Asia
				rs1412444	9	intergenic	ABO; SURF6	2365	2678	1.26	China	Asia
				rs10952541	19	intronic	SMARCA4	2365	2678	1.01	China	Asia
				rs3798220	6	missense	LPA	2365	2678	1.12	China	Asia
				rs579459	9	intergenic	ABO; SURF6	2365	2678	1.04	China	Asia
				rs1746048	10	intergenic	LINC00841; C10orf142	2365	2678	1.3	China	Asia
84	24489861	https://doi.org/10.1371/journal.pone.0087168	84	rs156019	5	ncRNA-intronic	LOC101929710	425	258	1.21	China	Asia
				rs6230	5	5'-UTR	PCSK1	425	258	1.14	China	Asia
				rs6233	5	synonymous	PCSK1	425	258	1.11	China	Asia
85	24505095	https://doi.org/10.1177/1470320313497819	85	rs4646994	17	intronic	ACE	141	369	2.08	Iran	Asia
				rs2010963	6	5'-UTR	VEGFA	141	369	1.75	Iran	Asia
86	24573017	https://doi.org/10.5551/jat.22640	86	rs12190287	1	intergenic	IL20; IL24	1503	no controls	1.52	Japan	Asia
				rs1333049	9	intergenic	CDKN2B-AS1; DMRTA1	1503	no controls	1.427	Japan	Asia
				rs4773144	13	intronic	COL4A2	1504	no controls	163	Japan	Asia
				rs46522	17	intronic	UBE2Z	1503	no controls	1.154	Japan	Asia
87	24599757	https://doi.org/10.1007/s12031-014-0238-2	87	rs505151	1	exonic	PCSK9	192	66	1.33	Tunisia	North Africa
				rs2230806	9	exonic	ABCA1	120	100	2.362	Saudi Arabia	Asia
88	24699044	https://doi.org/10.3390/ijms15045623	88	rs7529229	1	intronic	IL6R	1206	no controls	1.31	China	Asia
89	24776095	https://doi.org/10.1186/1476-511x-13-74	89	rs10455872	6	intronic	LPA	1394	no controls	2.02	Brazil	South America
				rs3798220	6	exonic	LPA	1394	no controls	1.09	Brazil	South America
90	24786211	https://doi.org/10.1016/j.gene.2014.04.064	90	rs1573949	3	downstream	GATA2	2386	2171	1.15	Saudi Arabia	Asia
91	24818816	https://doi.org/10.1089/gtmb.2013.0431	91	rs28362491	4	ncRNA-intronic	LOC105377621	636	616	1.805	Han China	Asia
				rs28362491	4	ncRNA-intronic	LOC105377622	437	356	3.192	Uyghur China	Asia
92	29581828	https://doi.org/10.18632/oncotarget.23491	92	rs2322864	2	intergenic	CXCR4; THSD7B	1200	1200	1.31	China	Asia
				rs2471859	2	intronic	CXCR4	1200	1200	1.38	China	Asia
				rs117600832	2	intronic	CXCR4	1200	1200	1.35	China	Asia
				rs2228014	2	exonic	CXCR4	1200	1200	1.98	China	Asia
93	29654172	https://doi.org/10.1042/bsr20171320	93	rs6458155	6	intergenic	HIVEP1; EDN1	525	675	1.36	China	Asia
				rs4145451	6	5' UTR	EDN1	525	675	1.216	China	Asia
				rs9369217	6	5' UTR	EDN1	525	675	1.12	China	Asia
				rs2070699	6	intronic	EDN1	525	675	1.058	China	Asia
94	29654577	https://doi.org/10.1007/s10528-018-9859-4	94	rs1870634	10	intergenic	LINC00841; C10orf142	155	112	3.16	Iran	Asia
95	29657680	https://doi.org/10.5001/omj.2018.23	95	rs187238	11	upstream	IL18	100	100	1.43	Iran	Asia
96	29681992	https://doi.org/10.1186/s12986-018-0266-y	96	rs330910	8	3'-UTR	PPP1R3B	556	617	1.08	China	Asia
				rs9949	8	3'-UTR	PPP1R3B	556	617	1.17	China	Asia
				rs12785	8	3'-UTR	PPP1R3B	556	617	1.16	China	Asia
				rs330915	8	3'-UTR	PPP1R3B	556	617	1.17	China	Asia
97	29728394	https://doi.org/10.1161/circgen.117.002034	97	rs247616	16	intergenic	HERPUD1; CETP	5740	no controls	1.11	Netherlands	Europe
				rs12720922	16	intronic	CETP	5741	no controls	1.08	Netherlands	Europe
98	29734056	https://doi.org/10.1016/j.cyto.2018.04.035	98	rs1800629	6	intronic	TNF	301	305	3.09	India	Asia
				rs361525	6	upstream	TNF	301	305	1.35	India	Asia
				rs3021097	1	intronic	IL-10	301	305	1.72	India	Asia
99	29786102	https://doi.org/10.1590/1678-4685-gmb-2017-0008	99	rs660339	11	exonic	UCP2	948	763	1.15	Mexico	North America
				rs659366	11	upstream	UCP2	948	763	1.72	Mexico	North America
				rs1800849	11	5' UTR	UCP3	943	763	1.16	Mexico	North America
100	29789399	https://doi.org/10.1042/bsr20180324	100	rs987401919	5	5' UTR	EBF1	243	215	1.15	China	Asia
				rs36071027	5	intronic	EBF1	243	215	1.531	China	Asia

101	29843469	https://doi.org/10.3390/jcdd5020031	101	rs688	19	exonic	LDLR	200	200	3	India	Asia
102	29866721	https://doi.org/10.1136/bmjopen-2017-020016	102	rs662799	11	upstream	APOA5	355	355	1.21	China	Asia
				rs651821	11	5' UTR	APOA5	355	355	1.57	China	Asia
				rs2075291	11	missense	APOA5	355	355	1.11	China	Asia
103	29960587	https://doi.org/10.1186/s12199-018-0719-y	103	rs671	12	exonic	ALDH2	161	495	1.72	China	Asia
104	29963349	https://doi.org/10.1007/s13205-018-1312-1	104	rs325400	15	exonic	MEF2A	120	100	2.01	Saudi Arabia	Asia
105	30011860	https://doi.org/10.3390/jcdd5030038	105	rs4680	22	exonic	COMT	100	100	3.32	Saudi Arabia	Asian
106	30024021	https://doi.org/10.1111/ahg.12273	106	rs3135506	11	exonic	APOA5	470	537	2.18	America	North America
107	30038730	https://doi.org/10.5001/omj.2018.57	107	rs2422493	9	upstream	ABCA1	110	110	2.28	Iran	Asia
108	30072947	https://doi.org/10.3389/fendo.2018.00362	108	rs4987574	7	intergenic	TRPV6; TRPV5	865	927	6.03	China	Asia
				rs1333049	9	intergenic	CDKN2B-AS1; DMRTA1	865	927	6.03	China	Asia
				rs2383207	9	ncRNA-intronic	CDKN2B-AS1	865	927	6.03	China	Asia
				rs1333040	9	ncRNA-intronic	CDKN2B-AS1	865	927	6.03	China	Asia
109	30073578	https://doi.org/10.1007/s10528-018-9880-7	109	rs4073	4	upstream	CXCL8	500	500	3.121	India	Asia
				rs1800872	1	upstream	IL10	500	500	4.106	India	Asia
110	30086706	https://doi.org/10.1186/s12872-018-0905-2	110	rs7396366	11	intronic	AP2A2	335	372	1.364	China	Asia
111	30181378	https://doi.org/10.1042/bsr20180839	111	rs2576178	10	upstream	RNLS	446	507	1.33	China	Asia
112	30271603	https://doi.org/10.3892/br.2018.1121	112	rs1042522	17	exonic	TP53	70	83	1.66	Iran	Asia
113	30289085	https://doi.org/10.2174/1871530318666181005095724	113	rs6505162	17	ncRNA-exonic	MIR423	100	117	2.02	India	Asia
114	30526064	https://doi.org/10.1089/gtmb.2018.0227	114	rs2781667	6	upstream	ARG1	240	240	2.65	Pakistan	Asia
115	30555085	https://doi.org/10.1007/s12041-018-1043-4	115	rs505151	1	exonic	PCSK9	500	500	1.59	India	Asia
116	30560371	https://doi.org/10.1007/s00011-018-1206-z	116	rs11614913	12	ncRNA-exonic	MIR196A2	218	611	1.76	Mexico	North America
117	30563176	https://doi.org/10.3390/biom8040164	117	rs528732638	18	intergenic	AQP4-AS1	78	264	3.62	Germany	Europe
118	30568542	https://doi.org/10.1515/jomb-2017-0005	118	rs2292832	2	ncRNA-exonic	MIR149	272	149	1.2	Iran	Asia
119	30581332	https://doi.org/10.1515/jomb-2017-0015	119	rs4588	4	exonic	GC	143	145	1.42	Iran	Asia
120	30595311	https://doi.org/10.1016/j.ihj.2018.06.016	120	rs7025486	9	intronic	DAB2IP	214	125	1.157	India	Asia
121	30647679	https://doi.org/10.1016/j.jgeb.2017.08.002	121	rs1801282	3	exonic	PPARG	100	100	1.6	Egypt	Africa, Asia
122	30662365	https://doi.org/10.1155/2018/1847696	122	rs9371533	6	exonic	RAET1E	1158	1104	1.255	Mexico	North America
				rs9383921	6	exonic	RAET1E	1158	1104	1.232	Mexico	North America
				rs7756850	6	ncRNA-intronic	RAET1E-AS1	1158	1104	1.284	Mexico	North America
				rs6925151	6	exonic	RAET1E	1158	1104	1.259	Mexico	North America
123	28743890	https://doi.org/10.1038/s41598-017-06732-9	123	rs11615	19	exonic	ERCC1	806	816	1.23	China	Asia
124	19171343	https://doi.org/10.1016/j.atherosclerosis.2008.12.026	124	rs1333049	9	intergenic	CDKN2B-AS1; DMRTA1	593	no controls	1.03	China	Asia
125	19197259	https://doi.org/10.1038/oby.2008.669	125	rs7566605	2	intergenic	CCDC93; I NSIG2	1460	1215	1.01	China	Asia
126	19435423	https://doi.org/10.1089/dna.2009.0866	126	rs3918242	20	intronic	MMP9	146	122	1.55	China	Asia
				rs2070744	7	intronic	NOS3	146	122	1.32	China	Asia
				rs1799983	7	missense	NOS3	146	122	1.15	Finland	Europe
127	20111910	https://doi.org/10.1007/s11033-010-9951-2	127	rs2228314	22	missense	SREBF2	197	234	1.247	Finland	Europe
128	20167083	https://doi.org/10.1186/1471-2350-11-28	128	rs2066865	4	intronic	FGG	305	305	1.38	Greece	Europe
				rs2070006	4	5'-UTR	FGA	305	305	1.32	Greece	Europe
129	20230296	https://doi.org/10.1089/dna.2009.0987	129	rs1129055	3	missense	CD86	164	299	1.07	Europe	Europe
130	21062467	https://doi.org/10.1186/1471-2350-11-157	130	rs2259816	12	intronic	HNF1A	665	2448	1.026	Durham	North America
131	21257319	https://doi.org/10.1016/j.cyto.2010.12.018	131	rs4950928	1	5'-UTR	CHI3L1	213	248	1.062	Washington	North America
				rs10399931	1	5'-UTR	CHI3L2	213	248	1.062	Washington	North America
				rs10399805	1	5'-UTR	CHI3L2	213	248	1.062	Washington	North America
132	23141087	https://doi.org/10.3760/cma.j.issn.0253-3758.2012.09.007	132	rs60231678	14	intergenic	SERPINA12; SERPINA4	776	794	1.05	China	Asia
				rs2236242	14	intronic	SERPINA12	776	794	1.32	China	Asia
133	23545315	https://doi.org/10.1016/j.gene.2013.03.091	133	rs699947	6	intronic	VEGFA	242	253	1.665	China	Asia
				rs2010963	6	5'-UTR	VEGFA	242	253	1.611	China	Asia
				rs833068	6	intronic	VEGFA	242	253	1.182	China	Asia

				rs3025000	6	intronic	VEGFA	242	253	1.126	China	Asia
				rs3025010	6	intronic	VEGFA	242	253	1.291	China	Asia
134	23631657	https://doi.org/10.1111/ene.12183	134	rs646776	1	downstream	CELSR2	2250	930	1.05	Sweden	Europe
				rs17609940	6	intronic	ANKS1A	2250	930	1.04	Sweden	Europe
				rs12190287	6	3'-UTR	TCF21	2250	930	1.01	Sweden	Europe
				rs3798220	6	missense	LPA	2250	930	1.07	Sweden	Europe
				rs4977574	9	intronic	CDKN2B-AS1	2250	930	1.12	Sweden	Europe
				rs579459	9	intergenic	ABO; SURF6	2250	930	1.07	Sweden	Europe
				rs1746048	10	intergenic	LINC00841; C10orf142	2250	930	1.06	Sweden	Europe
				rs964184	11	3' UTR	ZPR1	2250	930	1.11	Sweden	Europe
				rs2895811	14	intronic	HHIPL1	2250	930	1.02	Sweden	Europe
				rs3825807	15	missense	ADAMTS7	2250	930	1.01	Sweden	Europe
				rs12936587	17	intergenic	PEMT; SMCR2	2250	930	1.01	Sweden	Europe
				rs46522	17	intronic	UBE2Z	2250	930	1.01	Sweden	Europe
				rs1122608	19	intronic	SMARCA4	2250	930	1.05	Sweden	Europe
				rs9982601	21	intergenic	LINC00310; KCNE2	2250	930	1.04	Sweden	Europe
135	23639961	https://doi.org/10.1016/j.gene.2013.04.048	135	rs659366	11	upstream	UCP2	100	100	1.588	India	Asia
				rs1800849	11	5'-UTR	UCP3	100	100	3.733	India	Asia
136	23794009	https://doi.org/10.1007/s12013-013-9704-7	136	rs2910164	5	ncRNA-exonic	MIR146A; MIR3142HG	106	100	1.025	India	South Africa
137	24052696	https://doi.org/10.2478/v10034-011-0011-6	137	rs1946518	11	intronic	AP002884.2	169	326	1.1	Iran	Asia
