# Supplementary material for: Association of coronary artery disease related single nucleotide-polymorphisms with extreme Prakriti types: Insights from a case control study
Source: J Ayurveda Integr Med. 2026 Jul 8;17(4):101371. doi: 10.1016/j.jaim.2026.101371 (PMC13356639; doi:10.1016/j.jaim.2026.101371)
Supplement: Supplementary file 2 — List of 255 susceptible polymorphisms with associated genes. Multimedia component. 2 [file mmc2.pdf]

Supplementary File 2

S. No.	rs-ID	Gene	
1	rs11568828	CDKN2B-AS1	Cyclin dependent kinase inhibitor 2B-antisense RNA 1
2	rs1883025	ABCA1	ATP binding cassette subfamily A member 1
3	rs2230806	ABCA1	ATP binding cassette subfamily A member 2
4	rs2422493	ABCA1	ATP binding cassette subfamily A member 3
5	rs11887534	ABCG8	ATP binding cassette subfamily G member 8
6	rs579459	ABO; SURF6	Alpha1-3-N-acetylgalactosaminyltransferase and alpha 1-3 galactosyltransferase
7	rs11274804	AC007254.3	Long non coding RNA
8	rs383830	AC068138.1 pseudogene	Long non coding RNA
9	rs4343	ACE	Angiotensin I converting enzyme
10	rs4340	ACE	Angiotensin I converting enzyme
11	rs4646994	ACE	Angiotensin I converting enzyme
12	rs3825807	ADAMTS7	ADAM metallopeptidase with thrombospondin type 1 motif 7
13	rs266729	ADIPOQ	Adiponectin, C1Q and collagen domain containing
14	rs182052	ADIPOQ	Adiponectin, C1Q and collagen domain containing
15	rs12495941	ADIPOQ	Adiponectin, C1Q and collagen domain containing
16	rs1063539	ADIPOQ	Adiponectin, C1Q and collagen domain containing
17	rs3821799	ADIPOQ-AS1	Adiponectin, C1Q and collagen domain containing antisense RNA 1
18	rs1800544	ADRA2	Adrenoceptor alpha 2
19	rs1801253	ADRB1	Adrenoceptor beta 1
20	rs4994	ADRB3	Adrenoceptor beta 3
21	rs6903956	ADTRP	Androgen dependent TFPI regulating protein
22	rs2070600	AGER	Advanced glycosylation end-product specific receptor
23	rs184003	AGER	Advanced glycosylation end-product specific receptor
24	rs1800625	AGER; PBX2	Advanced glycosylation end-product specific receptor
25	rs1800624	AGER; PBX2	Advanced glycosylation end-product specific receptor
26	rs671	ALDH2	Aldehyde dehydrogenase 2 family member
27	rs7217186	ALOX15	Arachidonate 15-lipoxygenase
28	rs2619112	ALOX15	Arachidonate 15-lipoxygenase
29	rs12762303	ALOX5	Arachidonate 5-lipoxygenase
30	rs2228064	ALOX5	Arachidonate 5-lipoxygenase

31	rs41526545	ALOX5	Arachidonate 5-lipoxygenase
32	rs2029253	ALOX5	Arachidonate 5-lipoxygenase
33	rs28395866	ALOX5	Arachidonate 5-lipoxygenase
34	rs2229136	ALOX5	Arachidonate 5-lipoxygenase
35	rs1132340	ALOX5	Arachidonate 5-lipoxygenase
36	rs4769055	ALOX55AP	Arachidonate 5-lipoxygenase activating protein
37	rs3803277	ALOX55AP	Arachidonate 5-lipoxygenase activating protein
38	rs3803278	ALOX55AP	Arachidonate 5-lipoxygenase activating protein
39	rs12721458	ALOX55AP	Arachidonate 5-lipoxygenase activating protein
40	rs10507391	ALOX5AP	Arachidonate 5-lipoxygenase activating protein
41	rs4769874	ALOX55AP	Arachidonate 5-lipoxygenase activating protein
42	rs9551963	ALOX55AP	Arachidonate 5-lipoxygenase activating protein
43	rs17216473	ALOX55AP	Arachidonate 5-lipoxygenase activating protein
44	rs9315050	ALOX55AP	Arachidonate 5-lipoxygenase activating protein
45	rs17609940	ANKS1A	Ankyrin repeat and sterile alpha motif domain containing 1A
46	rs1946518	AP002884.2	Long non coding RNA
47	rs7396366	AP2A2	Adaptor related protein complex 2 subunit alpha 2
48	rs651821	APOA5	Apolipoprotein A5
49	rs2075291	APOA5	Apolipoprotein A5
50	rs3135506	APOA5	Apolipoprotein A5
51	rs528732638	AQP4-AS1	Aquaporin antisense RNA 1
52	rs2781667	ARG1	Arginase 1
53	rs10761600	ARID5B	AT-rich interaction domain 5B
54	rs7087507	ARID5B	AT-rich interaction domain 5B
55	rs11066001	BRAP	BRCA1 associated protein
56	rs11206510	BSND; PCSK9	Barttin CLCNK type accessory subunit beta
57	rs2075650	BSND; PCSK9	Barttin CLCNK type accessory subunit beta
58	rs11212617	C11orf65	Chromosome 11 open reading frame 65
59	rs2043211	CARD8	Caspase recruitment domain family member 8
60	rs7521023	CASQ2	Calsequestrin 2
61	rs6665970	CASQ3	Calsequestrin 3
62	rs9428090	CASQ4	Calsequestrin 4

63	rs7566605	CCDC93; INSIG2	Coiled-Coil domain containing 93; Insulin induced gene 2
64	rs2857656	CCL2	C-C motif chemokine ligand 2
65	rs1883832	CD40	CD40 molecule
66	rs2259816	CD86	CD86 molecule
67	rs12740374	CDC123; CAMK1D	Cell division cycle protein 123 homolog; Calcium/calmodulin-dependent protein kinase ID
68	rs8055236	CDH13	Cadherin 13
69	rs2383207	CDKN2B-AS1	Cyclin dependent kinase inhibitor 2B-antisense RNA 1
70	rs2383206	CDKN2B-AS1	Cyclin dependent kinase inhibitor 2B-antisense RNA 1
71	rs1799864	CDKN2B-AS1	Cyclin dependent kinase inhibitor 2B-antisense RNA 1
72	rs4977574	CDKN2B-AS1	Cyclin dependent kinase inhibitor 2B-antisense RNA 1
73	rs1333040	CDKN2B-AS1	Cyclin dependent kinase inhibitor 2B-antisense RNA 1
74	rs1333049	CDKN2B-AS1; DMRTA1	Cyclin dependent kinase inhibitor 2B-antisense RNA 1; Doublesex and mab-3 related transcription factor like family A1
75	rs10757278	CDKN2B-AS1; DMRTA1	Cyclin dependent kinase inhibitor 2B-antisense RNA 1; Doublesex and mab-3 related transcription factor like family A1
76	rs10757274	CDKN2B-AS1; DMRTA1	Cyclin dependent kinase inhibitor 2B-antisense RNA 1; Doublesex and mab-3 related transcription factor like family A1
77	rs5498	CDKN2B-AS1; DMRTA1	Cyclin dependent kinase inhibitor 2B-antisense RNA 1; Doublesex and mab-3 related transcription factor like family A1
78	rs1994016	CDKN2B-AS1; DMRTA1	Cyclin dependent kinase inhibitor 2B-antisense RNA 1; Doublesex and mab-3 related transcription factor like family A1
79	rs10811661	CDKN2B-AS1; DMRTA1	Cyclin dependent kinase inhibitor 2B-antisense RNA 1; Doublesex and mab-3 related transcription factor like family A1
80	rs2383208	CDKN2B-AS1; DMRTA1	Cyclin dependent kinase inhibitor 2B-antisense RNA 1; Doublesex and mab-3 related transcription factor like family A1
81	rs12779790	CELSR2	Cadherin EGF LAG seven-pass G-type receptor 2
82	rs646776	CELSR2	Cadherin EGF LAG seven-pass G-type receptor 2
83	rs1864163	CETP	Cholesteryl ester transfer protein
84	rs1800775	CETP	Cholesteryl ester transfer protein
85	rs5882	CETP	Cholesteryl ester transfer protein
86	rs12720922	CETP	Cholesteryl ester transfer protein
87	rs10399931	CHI3L2	Chitinase 3 Like 2 proteins

88	rs10399805	CHI3L2	Chitinase 3 Like 2 proteins
89	rs12413409	CNNM2	Cyclin and CBS domain divalent metal cation transport mediator 2
90	rs565470	COL4A1	Collagen type IV alpha 1 chain
91	rs605143	COL4A1	Collagen type IV alpha 1 chain
92	rs4773144	COL4A1	Collagen type IV alpha 1 chain
93	rs4680	COMT	Catechol-O-methyltransferase
94	rs3767443	CREG1	Cellular repressor of E1A stimulated genes 1
95	rs3753921	CREG1	Cellular repressor of E1A stimulated genes 1
96	rs1801157	CXCL12	C-X-C motif chemokine ligand 12
97	rs3744700	CXCL16	C-X-C motif chemokine ligand 16
98	rs2304973	CXCL16, ZMYND15	Chorionic somatomammotropin hormone like 1; Zinc Finger MYND-Type Containing 15
99	rs4073	CXCL8	C-X-C motif chemokine ligand 8
100	rs2471859	CXCR4	C-X-C motif chemokine receptor 4
101	rs117600832	CXCR4	C-X-C motif chemokine receptor 4
102	rs2228014	CXCR4	C-X-C motif chemokine receptor 4
103	rs7025486	DAB2IP	DAB2 interacting protein
104	rs987401919	EBF1	EBF transcription factor 1
105	rs36071027	EBF1	EBF transcription factor 1
106	rs4145451	EDN1	Endothelin 1
107	rs9369217	EDN1	Endothelin 1
108	rs2070699	EDN1	Endothelin 1
109	rs11615	ERCC1	Excision repair 1, endonuclease non-catalytic subunit
110	rs174460	FADS3	Fatty acid desaturase 3
111	rs1129055	FGA	Fibrinogen alpha chain
112	rs2070006	FGA; FGG	Fibrinogen alpha chain; fibrinogen gamma chain
113	rs2066865	FGG	Fibrinogen gamma chain)
114	rs17672135	FMN2	Formin 2
115	rs2322864	GATA2	GATA binding protein 2
116	rs4588	GC	GC vitamin D binding protein
117	rs6171	GH1	Growth hormone 1
118	rs2005172	GH1	Growth hormone 1
119	rs2005171	GH1	Growth hormone 1

120	rs247616	HERPUD1; CETP	Homocysteine inducible ER protein with ubiquitin like domain
121	rs2895811	HHIPL1	HHIP like 1
122	rs6458155	HIVEP1; EDN1	HIVEP zinc finger 1; Endothelin 1
123	rs1470579	HMGCR	3-hydroxy-3-methylglutaryl-CoA reductase
124	rs4950928	HNF1A	HNF1 homeobox A
125	rs1800872	IL10	Interleukin 10
126	rs3021097	IL-10	Interleukin 10
127	rs8034928	IL16	Interleukin 16
128	rs11556218	IL16	Interleukin 16
129	rs3848180	IL16	Interleukin 16
130	rs187238	IL18	Interleukin 18
131	rs5744292	IL18	Interleukin 18
132	rs187238	IL18	Interleukin 18
133	rs7529229	IL6R	Interleukin 6 receptor
134	rs1799963	ITGA2	Integrin subunit alpha 2
135	rs1126643	ITGA2	Integrin subunit alpha 2
136	rs13075202	KALRN	Kalirin RhoGEF kinase
137	rs1444768	KALRN	Kalirin RhoGEF kinase
138	rs1444754	KALRN	Kalirin RhoGEF kinase
139	rs4234218	KALRN	Kalirin RhoGEF kinase
140	rs2283228	KCNQ1	Potassium voltage-gated channel subfamily Q member 1
141	rs2237897	KCNQ1	Potassium voltage-gated channel subfamily Q member 1
142	rs2237895	KCNQ1	Potassium voltage-gated channel subfamily Q member 1
143	rs7157492	PRORP	Protein only RNase P Catalytic subunit
144	rs4981283	KIAA0391	Low density lipoprotein receptor
145	rs688	LDLR	Low density lipoprotein receptor
146	rs662799	LDLR	Low density lipoprotein receptor
147	rs9982601	LINC00310; KCNE2	Long intergenic non-protein coding RNA 310
148	rs501120	LINC00841; C10orf142	Long intergenic non-protein coding RNA 841
149	rs1537378	LINC00841; C10orf142	Long intergenic non-protein coding RNA 841
150	rs3846663	LINC00841; C10orf142	Long intergenic non-protein coding RNA 841
151	rs1746048	LINC00841; C10orf142	Long intergenic non-protein coding RNA 841

152	rs1870634	LINC00841; C10orf142	LINC00841 (long intergenic non-protein coding RNA 841
153	rs2246833	LIPA	Lipase A, lysosomal acid type
154	rs1412444	LIPA	Lipase A, lysosomal acid type
155	rs6507931	LIPG	Lipase G, endothelial type
156	rs156019	LOC101929710	Uncharacterized LOC101929710
157	rs7802307	LOC541472	Uncharacterized LOC646736)
158	rs10455872	LPA	Lipoprotein(a)
159	rs3798220	LPA	Lipoprotein(a)
160	rs3798220	LPA	Lipoprotein(a)
161	rs1573949	LPA	Lipoprotein(a)
162	rs28362491	LPA	Lipoprotein(a)
163	rs17222814	LTA4H	Leukotriene A4 hydrolase
164	rs34851361	MEF2A	Myocyte enhancer factor 2A
165	rs1059759	MEF2A	Myocyte enhancer factor 2A
166	rs325400	MEF2A	Myocyte enhancer factor 2A
167	rs2943634	MIA3	MIA SH3 domain ER export factor 3
168	rs17465637	MIA3	MIA SH3 domain ER export factor 3
169	rs10757283	MIA3	MIA SH3 domain ER export factor 3
170	rs2910164	MIR146A	MicroRNA 146a
171	rs2292832	MIR149	MicroRNA 1469
172	rs11614913	MIR196A2	MicroRNA 196A2
173	rs6505162	MIR423	MicroRNA 423
174	rs3812316	MLXIPL	MLX interacting protein like
175	rs3918242	MMP9	Matrix metallopeptidase 9
176	rs699947	MRPS18A; VEGFA	Mitochondrial ribosomal protein S18A,
177	rs13306541	MSR1	Macrophage scavenger receptor 1
178	rs416748	MSR1	Macrophage scavenger receptor 1
179	rs10903323	MSRA	Methionine sulfoxide reductase A
180	rs6922269	MTHFD1L	Methylenetetrahydrofolate dehydrogenase (NADP+ dependent) 1 like
181	rs1801133		Methylenetetrahydrofolate reductase
182	rs4846049	MTHFR	Methylenetetrahydrofolate reductase
183	rs9770242	NAMPT	Nicotinamide phosphoribosyl transferase

184	rs2070744	NOS3	Nitric oxide synthase 3
185	rs1799983	NOS3	Nitric oxide synthase 3
186	rs 6025	P2RY12	Purinergic receptor P2Y12
187	rs6785930	P2RY12	Purinergic receptor P2Y12
188	rs6230	PCSK1	Proprotein convertase subtilisin/kexin type 1
189	rs6233	PCSK1	Proprotein convertase subtilisin/kexin type 1
190	rs505151	PCSK9	Proprotein convertase subtilisin/kexin type 9
191	rs12936587	PEMT; SMCR2	Phosphatidylethanolamine N-Methyltransferase; Smith-Magenis syndrome chromosome
192	rs662	PON1	Paraoxonase 1
193	rs1801282	PPARG	Peroxisome proliferator activated receptor gamma
194	rs330910	PPP1R3B	Protein phosphatase 1 regulatory subunit 3B
195	rs9949	PPP1R3B	Protein phosphatase 1 regulatory subunit 3B
196	rs12785	PPP1R3B	Protein phosphatase 1 regulatory subunit 3B
197	rs330915	PPP1R3B	Protein phosphatase 1 regulatory subunit 3B
198	rs3805486	PRKAA1	Protein kinase AMP-activated catalytic subunit alpha 1
199	rs13361707	PRKAA1	Protein kinase AMP-activated catalytic subunit alpha 1
200	rs1048990	PSMA6	Proteasome 20S Subunit Alpha 6
201	rs599839	PSRC1	Proline and serine rich coiled-coil 1
202	rs1613662	PTGS1	Prostaglandin-Endoperoxide Synthase 1
203	rs6730157	RAB3GAP1	RAB3 GTPase activating protein catalytic subunit 1
204	rs9371533	RAET1E	Retinoic acid early transcript 1E
205	rs9383921	RAET1E	Retinoic acid early transcript 1E
206	rs6925151	RAET1E	Retinoic acid early transcript 1E
207	rs7756850	RAET1E-AS1	Retinoic acid early transcript 1E; Antisense RNA 1
208	rs9838682	RBMS3; LINC01985	RNA binding motif single stranded interacting protein 3; long intergenic non-protein coding RNA 1985
209	rs2576178	RNLS	Renalase, FAD dependent amine oxidase
210	rs6810298	ROPN1	Rhophilin associated tail protein 1
211	rs17376453	ROPN1	Rhophilin associated tail protein 1
212	rs7434266*	ROPN1	Rhophilin associated tail protein 1
213	rs9289231	ROPN1; KALRN	Rhophilin associated tail protein 1; Kalirin rhoGEF kinase
214	rs7613868	ROPN1; KALRN	Rhophilin associated tail protein 1; Kalirin rhoGEF kinase

215	rs12634530	ROPN1; KALRN	Rhophilin associated tail protein1; Kalirin rhoGEF kinase
216	rs12637456	ROPN1; KALRN	Rhophilin associated tail protein 1; Kalirin rhoGEF kinase
217	rs5888	SCARB1	Scavenger receptor class B member 1
218	rs699947	SERPINA12	Serpin family A member 12
219	rs60231678	SERPINA12; SERPINA4	Serpin family A member 12; (serpin family A member)
220	rs2236242	SERPINA12	Serpin family A member 12
221	rs3008621	SMAD3	Mothers against decapentaplegic homolog 3
222	rs1122608	SMARCA4	SWI/SNF related, matrix associated, actin dependent regulator of chromatin, subfamily a, member 4
223	rs10952541	SMARCA4	SWI/SNF related, matrix associated, actin dependent regulator of chromatin, subfamily a, member 4
224	rs2228314	SREBF2	Sterol regulatory element binding transcription factor 2
225	rs12190287	TCF21	Transcription factor 21
226	rs7903146	TCF7L2	Transcription factor 7 like 2
227	rs1800629	TNF	Tumor necrosis factor
228	rs361525	TNF	Tumor necrosis factor
229	rs5029930	TNFAIP3	TNF alpha induced protein 3
230	rs610604	TNFAIP3	TNF alpha induced protein 3
231	rs583522	TNFAIP3	TNF alpha induced protein 3
232	r5029933	TNFAIP3	TNF alpha induced protein 3
233	rs1042522	TP53	Tumor protein p53
234	rs4987574	TRPV6; TRPV5	Transient receptor potential V6; Transient receptor potential V5
235	rs7961581	TSPAN8; LGR5	Tetra spanin8; leucine-rich repeat-containing receptor5
236	rs46522	UBE2Z	Ubiquitin conjugating enzyme E2Z
237	rs660339	UCP2	Uncoupling protein 2
238	rs659366	UCP2	Uncoupling protein 2
239	rs1800849	UCP2	Uncoupling protein 2
240	rs2228570	VDR	Vitamin D receptor gene
241	rs731236	VDR	Vitamin D receptor gene
242	rs7975232	VDR	Vitamin D receptor gene
243	rs1544410	VDR	Vitamin D receptor gene
244	rs3025039	VEGFA	Vascular endothelial growth factor A

245	rs2010963	VEGFA	Vascular endothelial growth factor A
246	rs2010963	VEGFA	Vascular endothelial growth factor A
247	rs833068	VEGFA	Vascular endothelial growth factor A
248	rs3025000	VEGFA	Vascular endothelial growth factor A
249	rs3025010	VEGFA	Vascular endothelial growth factor A
250	rs25487	XRCC1	X-ray repair cross complementing 1
251	rs1799782	XRCC1	X-ray repair cross complementing 1
252	rs861539	XRCC3	X-ray repair cross complementing 3
253	rs3918242	ZNF335; MMP9	Zinc finger protein 335; Matrix metalloproteinase-9
254	rs2077316	ZNF365	Zinc finger protein 365
255	rs964184	ZPR1	ZPR1 zinc finger
