# Supplementary material for: Association of coronary artery disease related single nucleotide-polymorphisms with extreme Prakriti types: Insights from a case control study
Source: J Ayurveda Integr Med. 2026 Jul 8;17(4):101371. doi: 10.1016/j.jaim.2026.101371 (PMC13356639; doi:10.1016/j.jaim.2026.101371)
Supplement: Supplementary file 3 — Details of the data for calculation of cumulative Odds Ratio. Multimedia component. 3 [file mmc3.pdf]

Supplementary File 3

Variable: SNPs (In the present study, the variable under consideration is SNP and not the studies)

Variable for estimate: Odds Ratio (OR)

Variable for standard error: Standard Error

Generic inverse variance method was used to calculate the pooled OR				
S. No.	347 Individual SNPs from studies with OR>1	Estimate	Standard Error	95% CI
1	rs5029930	2.3	1.586	-0.809 to 5.409
2	rs610604	2.4	1.839	-1.204 to 6.004
3	rs583522	1.7	1.139	-0.532 to 3.932
4	rs610604	1.1	0.6	-0.0760 to 2.276
5	rs5029930	1.4	0.89	-0.344 to 3.144
6	r5029933	1.8	1.254	-0.658 to 4.258
7	rs9289231	3.1	2.345	-1.496 to 7.696
8	rs6810298	1.7	1.139	-0.532 to 3.932
9	rs17376453	2.1	1.539	-0.916 to 5.116
10	rs7434266	1.8	1.254	-0.658 to 4.258
11	rs7613868	2.6	1.999	-1.318 to 6.518
12	rs12634530	1.3	0.841	-0.348 to 2.948
13	rs12637456	2.2	1.588	-0.912 to 5.312
14	rs13075202	2.1	1.539	-0.916 to 5.116
15	rs1444768	1.8	1.254	-0.658 to 4.258
16	rs1444754	1.7	1.144	-0.542 to 3.942
17	rs4234218	1.4	0.89	-0.344 to 3.144
18	rs599839	1.13	0.716	-0.273 to 2.533
19	rs3008621	1.1	0.6	-0.0760 to 2.276
20	rs2943634	1.2	0.659	-0.0916 to 2.492
21	rs501120	1.11	0.6	-0.0660 to 2.286
22	rs6922269	1.23	0.653	-0.0499 to 2.510

23	rs1333049	1.37	0.727	-0.0549 to 2.795
24	rs1333049	1.33	0.728	-0.0969 to 2.757
25	rs1333049	1.44	0.863	-0.251 to 3.131
26	rs17222814	1.5	0.995	-0.450 to 3.450
27	rs10507391	1.2	0.726	-0.223 to 2.623
28	rs4769874	1.23	0.883	-0.501 to 2.961
29	rs9551963	1.22	0.735	-0.221 to 2.661
30	rs17216473	1.35	0.865	-0.345 to 3.045
31	rs9315050	1.05	0.718	-0.357 to 2.457
32	rs12762303	1.32	0.8	-0.248 to 2.888
33	rs12762303	1.46	0.919	-0.341 to 3.261
34	rs12762303	1.29	0.805	-0.288 to 2.868
35	rs12762303	1.22	0.726	-0.203 to 2.643
36	rs12762303	1.19	0.649	-0.0820 to 2.462
37	rs2228064	1.24	0.741	-0.212 to 2.692
38	rs41526545	1.1	0.69	-0.252 to 2.452
39	rs41526545	1.27	0.755	-0.210 to 2.750
40	rs41526545	1.56	0.968	-0.337 to 3.457
41	rs2029253	1.35	0.865	-0.345 to 3.045
42	rs2029253	1.84	1.229	-0.569 to 4.249
43	rs2029253	1.31	0.805	-0.268 to 2.888
44	rs2029253	1.03	0.627	-0.199 to 2.259
45	rs28395866	1.27	0.755	-0.210 to 2.750
46	rs28395866	1.21	0.69	-0.142 to 2.562
47	rs28395866	1.04	0.618	-0.171 to 2.251
48	rs28395866	1.03	0.619	-0.183 to 2.243
49	rs2229136	1.12	0.6	-0.0560 to 2.296
50	rs2229136	1.32	0.8	-0.248 to 2.888
51	rs2229136	1.03	0.627	-0.199 to 2.259
52	rs4769055	1.34	1.054	-0.726 to 3.406
53	rs4769055	1.18	0.751	-0.292 to 2.652
54	rs10507391	1.17	0.639	-0.0824 to 2.422

55	rs10507391	1.03	0.627	-0.199 to 2.259
56	rs3803277	1.38	1.028	-0.635 to 3.395
57	rs3803277	1.13	0.741	-0.322 to 2.582
58	rs3803277	1.03	0.618	-0.181 to 2.241
59	rs3803278	1.05	0.718	-0.357 to 2.457
60	rs3803278	1.38	1.017	-0.613 to 3.373
61	rs3803278	1.07	0.616	-0.137 to 2.277
62	rs3803278	1.04	0.718	-0.367 to 2.447
63	rs12721458	1.14	0.741	-0.312 to 2.592
64	rs1132340	1.48	0.858	-0.202 to 3.162
65	rs7802307	3.676	2.819	-1.849 to 9.201
66	rs2383207	1.631	0.997	-0.323 to 3.585
67	rs10757278	1.955	1.191	-0.379 to 4.289
68	rs2383206	1.67	0.976	-0.243 to 3.583
69	rs2383207	1.72	1.001	-0.242 to 3.682
70	rs10757274	1.78	1.04	-0.258 to 3.818
71	rs10757278	1.76	1.03	-0.259 to 3.779
72	rs10761600	2.2	1.542	-0.822 to 5.222
73	rs7087507	1.82	1.274	-0.677 to 4.317
74	rs4343	1.56	0.968	-0.337 to 3.457
75	rs4340	1.795	1.111	-0.383 to 3.973
76	rs1333049	1.87	1.135	-0.355 to 4.095
77	rs17465637	1.45	0.863	-0.241 to 3.141
78	rs1333049	1.47	0.883	-0.261 to 3.201
79	rs501120	1.22	0.72	-0.191 to 2.631
80	rs2383206	1.69	0.828	0.0671 to 3.313
81	rs2383206	1.99	1.393	-0.740 to 4.720
82	rs2383206	1.39	0.82	-0.217 to 2.997
83	rs5498	4.8	0.807	3.218 to 6.382
84	rs10757278	1.11	0.595	-0.0562 to 2.276
85	rs1799864	6.14	5.211	-4.074 to 16.354
86	rs2857656	2.09	1.585	-1.017 to 5.197

87	rs1333049	1.48	0.858	-0.202 to 3.162
88	rs2228570	1.23	0.873	-0.481 to 2.941
89	rs3812316	2.96	2.297	-1.542 to 7.462
90	rs2283228	4.569	4.084	-3.436 to 12.574
91	rs2237897	1.569	1.243	-0.867 to 4.005
92	rs2237895	1.25	0.924	-0.561 to 3.061
93	rs1048990	1.18	0.751	-0.292 to 2.652
94	rs7157492	1.52	1	-0.440 to 3.480
95	rs4981283	1.08	0.641	-0.176 to 2.336
96	rs187238	3.08	2.842	-2.490 to 8.650
97	rs599839	1.28	0.636	0.0334 to 2.527
98	rs599839	1.09	0.805	-0.488 to 2.668
99	rs17465637	1.09	0.6	-0.0860 to 2.266
100	rs17465637	1.09	0.805	-0.488 to 2.668
101	rs17465637	1.09	0.6	-0.0860 to 2.266
102	rs17672135	1.11	0.601	-0.0680 to 2.288
103	rs2943634	1.12	0.64	-0.134 to 2.374
104	rs2943635	1.05	0.618	-0.161 to 2.261
105	rs383830	1.09	0.6	-0.0860 to 2.266
106	rs501120	1.09	0.6	-0.0860 to 2.266
107	rs501120	1.11	0.6	-0.0660 to 2.286
108	rs501120	1.09	0.595	-0.0762 to 2.256
109	rs8055236	1.04	0.596	-0.128 to 2.208
110	rs11066001	1.65	0.895	-0.104 to 3.404
111	rs11066001	1.68	1.619	-1.493 to 4.853
112	rs11066001	1.63	1.619	-1.543 to 4.803
113	rs34851361	1.22	0.761	-0.272 to 2.712
114	rs1059759	1.21	0.69	-0.142 to 2.562
115	rs1613662	1.88	1.355	-0.776 to 4.536
116	rs6025	1.718	1.432	-1.089 to 4.525
117	rs1799963	1.019	0.92	-0.784 to 2.822
118	rs6785930	1.005	0.707	-0.381 to 2.391

119	rs1126643	1.169	0.826	-0.450 to 2.788
120	rs10757274	1.52	0.949	-0.340 to 3.380
121	rs7903146	1.325	0.82	-0.282 to 2.932
122	rs3744700	1.77	1.117	-0.419 to 3.959
123	rs2304973	1.02	0.622	-0.199 to 2.239
124	rs9838682	1.66	1.619	-1.513 to 4.833
125	rs10455872	1.7	0.94	-0.142 to 3.542
126	rs3798220	1.92	1.165	-0.363 to 4.203
127	rs11274804	2.02	1.357	-0.640 to 4.680
128	rs2070006	1.51	0.995	-0.440 to 3.460
129	rs671	1.85	1.146	-0.396 to 4.096
130	rs6507931	1.09	0.595	-0.0762 to 2.256
131	rs11206510	1.49	0.944	-0.360 to 3.340
132	rs11887534	2.44	1.928	-1.339 to 6.219
133	rs7217186	2.6	1.999	-1.318 to 6.518
134	rs2619112	1.2	0.76	-0.290 to 2.690
135	rs1333049	1.524	0.916	-0.271 to 3.319
136	rs1994016	1.14	1.1	-1.016 to 3.296
137	rs10757278	1.17	0.639	-0.0824 to 2.422
138	rs688	1.67	1.109	-0.504 to 3.844
139	rs10757278	2.2	1.67	-1.073 to 5.473
140	rs3767443	1.27	0.852	-0.400 to 2.940
141	rs3753921	1.51	1.03	-0.509 to 3.529
142	rs1883832	2.779	2.052	-1.243 to 6.801
143	rs1883832	1.554	1.341	-1.074 to 4.182
144	rs1042522	2	1.321	-0.589 to 4.589
145	rs17465637	1.12	0.579	-0.0148 to 2.255
146	rs10757283	1.18	0.639	-0.0724 to 2.432
147	rs10811661	1.19	0.649	-0.0820 to 2.462
148	rs2383208	1.11	0.6	-0.0660 to 2.286
149	rs11568828	3.13	1.14	0.896 to 5.364
150	rs6171	3.13	1.14	0.896 to 5.364

151	rs2005172	3.13	1.14	0.896 to 5.364
152	rs2005171	3.13	1.14	0.896 to 5.364
153	rs10757274	1.8	1.183	-0.519 to 4.119
154	rs2236242	1.32	0.764	-0.177 to 2.817
155	rs8034928	1.43	0.818	-0.173 to 3.033
156	rs3848180	1.47	0.878	-0.251 to 3.191
157	rs6903956	2.03	1.499	-0.908 to 4.968
158	rs731236	1.75	1.219	-0.639 to 4.139
159	rs7975232	1.47	0.888	-0.270 to 3.210
160	rs1544410	1.4	0.977	-0.515 to 3.315
161	rs174460	1.896	1.298	-0.648 to 4.440
162	rs13306541	1.97	1.332	-0.641 to 4.581
163	rs416748	1.8	1.157	-0.468 to 4.068
164	rs266729	1.64	1.079	-0.475 to 3.755
165	rs182052	1.18	0.68	-0.153 to 2.513
166	rs12495941	1.04	0.596	-0.128 to 2.208
167	rs1063539	1.06	0.616	-0.147 to 2.267
168	rs3821799	1.07	0.616	-0.137 to 2.277
169	rs6730157	1.6	0.886	-0.137 to 3.337
170	rs2077316	2.41	1.828	-1.173 to 5.993
171	rs2043211	1.2	0.741	-0.252 to 2.652
172	rs662	3.2	2.486	-1.673 to 8.073
173	rs1333049	8.16	5.004	-1.648 to 17.968
174	rs17576	2.005	0.924	0.194 to 3.816
175	rs10903323	1.31	0.805	-0.268 to 2.888
176	rs3025039	1.26	0.909	-0.522 to 3.042
177	rs2010963	1.02	0.632	-0.219 to 2.259
178	rs8034928	2	1.438	-0.818 to 4.818
179	rs11556218	2.41	1.853	-1.222 to 6.042
180	rs9770242	1.46	0.919	-0.341 to 3.261
181	rs9770242	1.02	0.632	-0.219 to 2.259
182	rs5744292	1.87	1.708	-1.478 to 5.218

183	rs3918242	1.87	1.748	-1.556 to 5.296
184	rs2246833	1.41	1.112	-0.770 to 3.590
185	rs1412444	1.6	1.018	-0.395 to 3.595
186	rs7961581	1.26	0.745	-0.200 to 2.720
187	rs11206510	1.28	0.765	-0.219 to 2.779
188	rs2075650	1.14	0.737	-0.305 to 2.585
189	rs12740374	1.11	0.661	-0.186 to 2.406
190	rs12779790	1.03	0.627	-0.199 to 2.259
191	rs3846663	1.08	0.626	-0.147 to 2.307
192	rs1883025	1.04	0.611	-0.158 to 2.238
193	rs1470579	1.04	0.614	-0.163 to 2.243
194	rs1864163	1.16	0.737	-0.285 to 2.605
195	rs2383207	1.11	0.636	-0.137 to 2.357
196	rs1470579	1.04	0.606	-0.148 to 2.228
197	rs5888	1.76	1.229	-0.649 to 4.169
198	rs2070600	1.26	0.755	-0.220 to 2.740
199	rs184003	1.59	1.64	-1.624 to 4.804
200	rs1800625	1.75	1.224	-0.649 to 4.149
201	rs1800624	1.06	0.565	-0.0474 to 2.167
202	rs565470	1.993	1.341	-0.635 to 4.621
203	rs605143	1.369	0.848	-0.293 to 3.031
204	rs4994	1.7	1.139	-0.532 to 3.932
205	rs1801253	1.8	1.443	-1.028 to 4.628
206	rs1800544	1.1	0.982	-0.825 to 3.025
207	rs11212617	2.7	1.833	-0.893 to 6.293
208	rs1864163	1.16	0.803	-0.414 to 2.734
209	rs1800775	1.183	0.744	-0.275 to 2.641
210	rs5882	1.01	0.632	-0.229 to 2.249
211	rs25487	1.31	0.836	-0.329 to 2.949
212	rs1801133	1.54	0.989	-0.398 to 3.478
213	rs4846049	1.94	1.363	-0.731 to 4.611
214	rs861539	1.07	0.657	-0.218 to 2.358

215	rs1799782	1.53	0.938	-0.308 to 3.368
216	rs25487	1.11	0.621	-0.107 to 2.327
217	rs1800872	2.45	1.929	-1.331 to 6.231
218	rs3848180	1.79	1.229	-0.619 to 4.199
219	rs8034928	1.87	1.273	-0.625 to 4.365
220	rs1801157	2.28	1.515	-0.689 to 5.249
221	rs3805486	1.16	0.742	-0.294 to 2.614
222	rs13361707	1.17	0.721	-0.243 to 2.583
223	rs7521023	2.7	1.965	-1.151 to 6.551
224	rs6665970	1.4	0.931	-0.425 to 3.225
225	rs9428090	2.7	2.113	-1.441 to 6.841
226	rs2910164	1.39	0.75	-0.0800 to 2.860
227	rs11614913	1.05	0.621	-0.167 to 2.267
228	rs1122608	1.02	0.571	-0.0992 to 2.139
229	rs12190287	1.19	0.68	-0.143 to 2.523
230	rs12413409	1.12	0.61	-0.0756 to 2.316
231	rs4977574	1.5	0.832	-0.131 to 3.131
232	rs1412444	1.26	0.724	-0.159 to 2.679
233	rs10952541	1.01	0.546	-0.0602 to 2.080
234	rs3798220	1.12	0.829	-0.505 to 2.745
235	rs579459	1.04	0.627	-0.189 to 2.269
236	rs1746048	1.3	0.744	-0.158 to 2.758
237	rs156019	1.21	0.715	-0.191 to 2.611
238	rs6230	1.14	0.686	-0.205 to 2.485
239	rs6233	1.11	0.671	-0.205 to 2.425
240	rs4646994	2.08	1.751	-1.352 to 5.512
241	rs2010963	1.75	0.886	0.0134 to 3.487
242	rs12190287	1.52	0.661	0.224 to 2.816
243	rs1333049	1.427	1.061	-0.653 to 3.507
244	rs6903956	163	1.241	160.568 to 165.432
245	rs46522	1.154	0.68	-0.179 to 2.487
246	rs505151	1.33	2.599	-3.764 to 6.424

247	rs2230806	2.362	2.049	-1.654 to 6.378
248	rs7529229	1.31	1.028	-0.705 to 3.325
249	rs10455872	2.02	1.397	-0.718 to 4.758
250	rs3798220	1.09	0.816	-0.509 to 2.689
251	rs1573949	1.15	0.64	-0.104 to 2.404
252	rs28362491	1.805	1.263	-0.670 to 4.280
253	rs28362491	3.192	2.53	-1.767 to 8.151
254	rs2322864	1.31	0.902	-0.458 to 3.078
255	rs2471859	1.38	1.028	-0.635 to 3.395
256	rs117600832	1.35	1.069	-0.745 to 3.445
257	rs2228014	1.98	1.454	-0.870 to 4.830
258	rs6458155	1.36	0.86	-0.326 to 3.046
259	rs4145451	1.216	0.762	-0.278 to 2.710
260	rs9369217	1.12	0.773	-0.395 to 2.635
261	rs2070699	1.058	0.673	-0.261 to 2.377
262	rs1870634	3.16	2.502	-1.744 to 8.064
263	rs187238	1.43	1.012	-0.554 to 3.414
264	rs330910	1.08	0.636	-0.167 to 2.327
265	rs9949	1.17	0.685	-0.173 to 2.513
266	rs12785	1.16	0.691	-0.194 to 2.514
267	rs330915	1.17	0.685	-0.173 to 2.513
268	rs247616	1.11	0.584	-0.0346 to 2.255
269	rs12720922	1.08	0.565	-0.0274 to 2.187
270	rs1800629	3.09	1.113	0.909 to 5.271
271	rs361525	1.35	0.865	-0.345 to 3.045
272	rs3021097	1.72	1.001	-0.242 to 3.682
273	rs660339	1.15	0.64	-0.104 to 2.404
274	rs659366	1.72	0.843	0.0677 to 3.372
275	rs1800849	1.16	0.826	-0.459 to 2.779
276	rs987401919	1.15	1.308	-1.414 to 3.714
277	rs36071027	1.531	1.053	-0.533 to 3.595
278	rs688	3	2.32	-1.547 to 7.547

279	rs662799	1.21	1.054	-0.856 to 3.276
280	rs651821	1.57	1.5	-1.370 to 4.510
281	rs2075291	1.11	0.791	-0.440 to 2.660
282	rs671	1.72	2.224	-2.639 to 6.079
283	rs325400	2.01	1.326	-0.589 to 4.609
284	rs4680	3.32	4.923	-6.329 to 12.969
285	rs3135506	2.18	1.588	-0.932 to 5.292
286	rs2422493	2.28	3.172	-3.937 to 8.497
287	rs4987574	6.03	1.825	2.453 to 9.607
288	rs1333049	6.03	3.192	-0.226 to 12.286
289	rs2383207	6.03	2.247	1.626 to 10.434
290	rs1333040	6.03	1.72	2.659 to 9.401
291	rs4073	3.121	2.78	-2.328 to 8.570
292	rs1800872	4.106	3.02	-1.813 to 10.025
293	rs7396366	1.364	1.293	-1.170 to 3.898
294	rs2576178	1.33	1.054	-0.736 to 3.396
295	rs1042522	1.66	2.323	-2.893 to 6.213
296	rs6505162	2.02	1.7	-1.312 to 5.352
297	rs2781667	2.65	1.428	-0.149 to 5.449
298	rs505151	1.59	1.34	-1.036 to 4.216
299	rs11614913	1.76	0.891	0.0136 to 3.506
300	rs528732638	3.62	2.406	-1.096 to 8.336
301	rs2292832	1.2	1.349	-1.444 to 3.844
302	rs4588	1.42	1.772	-2.053 to 4.893
303	rs7025486	1.157	1.023	-0.848 to 3.162
304	rs1801282	1.6	1.192	-0.736 to 3.936
305	rs9371533	1.255	0.728	-0.172 to 2.682
306	rs9383921	1.232	0.716	-0.171 to 2.635
307	rs7756850	1.284	0.766	-0.217 to 2.785
308	rs6925151	1.259	0.749	-0.209 to 2.727
309	rs11615	1.23	0.716	-0.173 to 2.633
310	rs1333049	1.03	0.741	-0.422 to 2.482

311	rs7566605	1.01	0.626	-0.217 to 2.237
312	rs3918242	1.55	1.341	-1.078 to 4.178
313	rs2070744	1.32	1.061	-0.760 to 3.400
314	rs1799983	1.15	0.962	-0.736 to 3.036
315	rs2228314	1.247	1.29	-1.281 to 3.775
316	rs2066865	1.38	0.89	-0.364 to 3.124
317	rs2070006	1.32	0.954	-0.550 to 3.190
318	rs1129055	1.07	0.733	-0.367 to 2.507
319	rs2259816	1.026	0.579	-0.109 to 2.161
320	rs4950928	1.062	0.971	-0.841 to 2.965
321	rs10399931	1.062	0.807	-0.520 to 2.644
322	rs10399805	1.062	0.971	-0.841 to 2.965
323	rs60231678	1.05	0.621	-0.167 to 2.267
324	rs2236242	1.32	0.764	-0.177 to 2.817
325	rs699947	1.665	1.036	-0.366 to 3.696
326	rs2010963	1.611	1.619	-1.562 to 4.784
327	rs833068	1.182	0.714	-0.217 to 2.581
328	rs3025000	1.126	0.681	-0.209 to 2.461
329	rs3025010	1.291	0.805	-0.287 to 2.869
330	rs646776	1.05	0.56	-0.0476 to 2.148
331	rs17609940	1.04	0.555	-0.0478 to 2.128
332	rs12190287	1.01	0.551	-0.0700 to 2.090
333	rs3798220	1.07	0.677	-0.257 to 2.397
334	rs4977574	1.12	0.589	-0.0344 to 2.274
335	rs579459	1.07	0.57	-0.0472 to 2.187
336	rs1746048	1.06	0.575	-0.0670 to 2.187
337	rs964184	1.11	0.605	-0.0758 to 2.296
338	rs2895811	1.02	0.535	-0.0286 to 2.069
339	rs3825807	1.01	0.53	-0.0288 to 2.049
340	rs12936587	1.01	0.53	-0.0288 to 2.049
341	rs46522	1.01	0.53	-0.0288 to 2.049
342	rs1122608	1.05	0.57	-0.0672 to 2.167

343	rs9982601	1.04	0.56	-0.0576 to 2.138
344	rs659366	1.588	1.32	-0.999 to 4.175
345	rs1800849	3.733	3.26	-2.657 to 10.123
346	rs2910164	1.025	0.725	-0.396 to 2.446
347	rs1946518	1.1	0.843	-0.552 to 2.752
	Total (fixed effects)	1.503	0.0449	1.415 to 1.591
	**Total (random effects)**	**2.049**	**0.319**	**1.424 to 2.674**

Test for heterogeneity

Q	17057.3512
DF	347
Significance level	$p < 0.0001$
I^2 (inconsistency)	97.97%
95% CI for I^2	97.87 to 98.07

Publication bias

Egger's test	
Intercept	2.3185
95% CI	0.1488 to 4.4882
Significance level	$p = 0.0363$
Begg's test	
Kendall's Tau	0.7584
