# Supplementary material for: Association of coronary artery disease related single nucleotide-polymorphisms with extreme Prakriti types: Insights from a case control study
Source: J Ayurveda Integr Med. 2026 Jul 8;17(4):101371. doi: 10.1016/j.jaim.2026.101371 (PMC13356639; doi:10.1016/j.jaim.2026.101371)
Supplement: Supplementary file 4 — Details of 5 polymorphisms amongst 255 SNPs with significant association in the study population with details of 16 polymorphisms with p < 0.00001 in our population set and global allele frequencies of different populations from the 1000 Genomes Project and gnom AD database. [GC: Genome Correction; BON F: Bonferroni test; Sidak: Dunn-sidak-test; FDR: False Discovery Rate; AFR: African/African American; AMR: Admixed American; EAS: East Asians; SAS: South Asian; EUR: European; FIN: Finnish; NFE: Non-Finnish European; OTH: Other (Population not assigned)]. Multimedia component. 4 [file mmc4.pdf]

Supplementary File 5

																					Information from Indigen https://clingen.igib.res.in/indigen															
Results of Global Screening Array in our data set																					Minor allele frequency in different populations in 1000 Genome						Minor allele frequency in different populations in gnom AD									
S No.	Chromosome	rs-ID	GENE	Position	Base pairs	Reference allele	Frequency of alternate allele in cases	Frequency of alternate allele in controls	Alternate allele	Chi Square test	p Value	OR	UNADJ	GC	BONF	HOLM	SIDAK_SS	AMR	EAS	SAS	AMI	ASJ	FIN	NFE	OTH	EUR	All	AFR	AMR	EAS	SAS	AMI	ASJ	FIN	NFE	OTH
1	12	rs1544410	VDR	intronic	48239835	A	0.4851	0.2586	G	9.41	0.0022	2.701	0.002158	0.01007	0.199	0.199	0.1802	0.1802	0.1985	1	0.2959	0.2716	0.2565	0.0645	0.4847	0.4036	0.3505	0.305	0.3084	0.054	0.4435	0.2757	0.3623	0.3333	0.4022	0.351
2	15	rs3825807	ADAMTS7	exonic	79089111	G	0.2626	0.4483	A	7.298	0.0069	0.4384	0.006903	0.02344	0.635	0.628	0.4713	0.4676	0.3175	1	0.2502	0.1121	0.2968	0.1518	0.3241	0.4274	0.328	0.1582	0.3083	0.1545	0.3282	0.4081	0.4072	0.3454	0.4426	0.335
3	12	rs7975232	VDR	intronic	48238837	C	0.3861	0.569	A	6.156	0.0131	0.4765	0.01309	0.0374	1	1	0.7026	0.6946	0.3635	1	0.51538	0.6407	0.4409	0.2907	0.59	0.5547	0.5529	0.6259	0.5075	0.28	0.5565	0.5928	0.584	0.5517	0.5269	0.5358
4	1	rs1801133	MTHFR	exonic	11856378	A	0.1535	0.2963	G	5.799	0.016	0.4306	0.01603	0.04336	1	1	0.7739	0.7627	0.3635	1	0.24541	0.09	0.4741	0.2956	0.1186	0.3648	0.2719	0.1128	0.449	0.2873	0.1544	0.4822	0.4609	0.235	0.3352	0.3015
5	12	rs731236	VDR	exonic	48238757	G	0.3614	0.1964	A	5.433	0.0198	2.315	0.01975	0.05054	1	1	0.8405	0.8272	0.3635	1	0.27656	0.2852	0.255	0.0675	0.3691	0.3996	0.3393	0.2875	0.3049	0.0502	0.3252	0.2733	0.3593	0.3313	0.3964	0.3329
6	5	rs73796819	PPIC; LOC105379152	intergenic	1.22E+08	G	0.09314	0.3448	A	22.58	2.016E-06	0.1951	2.016E-06	2.701E-06	0.5041	0.5041	0.396	0.396	0.5041	1	0.23103	0.1906	0.3689	0.1726	0.1483	0.328	0.2785	0.1951	0.3364	0.1594	0.1598	0.3619	0.3396	0.2952	0.3241	0.303
7	11	rs2014365	PKNOX2	intronic	1.25E+08	G	0.1287	0.3966	A	21.14	4.278E-06	0.2248	4.278E-06	5.629E-06	1	1	0.657	0.657	0.535	1	0.28235	0.5575	0.0836	0.2778	0.2546	0.0895	0.2149	0.4897	0.1187	0.2974	0.2338	0.1033	0.1485	0.0593	0.0824	0.2069
8	8	rs16904078	CALB1; LINC00534	intergenic	91200774	C	0.02941	0.1897	A	19.11	1.233E-05	0.1295	1.233E-05	1.582E-05	1	1	0.9542	0.9542	0.7273	1	0.08367	0.1846	0.0533	0.0099	0.0573	0.0716	0.0946	0.1551	0.0593	0.0042	0.047	0.0611	0.0549	0.0738	0.0753	0.0918
9	22	rs135745	TMEM184B; CSNK1E	intergenic	38683637	G	0.549	0.2241	C	19.11	1.236E-05	4.214	1.236E-05	1.586E-05	1	1	0.9546	0.9546	0.7273	1	0.40715	0.3956	0.5533	0.1478	0.4652	0.5249	0.4756	0.3995	0.5377	0.1513	0.4559	0.5445	0.5331	0.5294	0.5158	0.4823
10	5	rs78607811	LOC105379152	ncRNA-intronic	1.22E+08	A	0.08824	0.3103	G	18.8	1.454E-05	0.2151	1.454E-05	1.858E-05	1	1	0.9737	0.9737	0.7273	1	0.13518	0.0605	0.1801	0.0327	0.1278	0.3121	0.2135	0.0849	0.2427	0.0323	0.1394	0.3133	0.2834	0.2584	0.2904	0.233
11	2	rs72813862	MIR217HG	ncRNA-intronic	56199810	A	0.1324	0.3793	G	18.11	0.0000208	0.2496	0.0000208	2.636E-05	1	1	0.9945	0.9945	0.7679	1	0.06929	0.0363	0.0548	0.0258	0.2055	0.0338	0.0428	0.0407	0.0523	0.0342	0.1869	0.0411	0.0346	0.0312	0.0384	0.034
12	12	rs7316141	A2ML1	intronic	9027429	G	0.2304	0.5172	A	17.91	2.321E-05	0.2794	2.321E-05	2.933E-05	1	1	0.997	0.997	0.7679	1	0.22165	0.1513	0.196	0.1746	0.3262	0.2773	0.2235	0.1583	0.2115	0.1641	0.2959	0.3105	0.4299	0.1546	0.2667	0.2372
13	1	rs2577032	USH2A	intronic	2.16E+08	A	0.14	0.3929	G	17.8	2.456E-05	0.2516	2.456E-05	0.000031	1	1	0.9979	0.9979	0.7679	1	0.81789	0.7239	0.8948	0.9147	0.7914	0.8171	0.7988	0.7315	0.833	0.9156	0.8076	0.773	0.7815	0.8017	0.8294	0.8031
14	17	rs7225835	MYH10	intronic	8431453	G	0.5147	0.2069	A	17.31	0.0000317	4.066	0.0000317	3.977E-05	1	1	0.9996	0.9996	0.881	1	0.49341	0.615	0.4539	0.501	0.4714	0.3748	0.4621	0.5599	0.4028	0.509	0.4419	0.3256	0.3383	0.4617	0.4188	0.4428
15	2	rs1275930	CIB4; KCNK3	intergenic	26870584	G	0.3088	0.6034	A	16.74	0.0000428	0.2936	0.0000428	5.332E-05	1	1	1	1	0.9301	1	0.45807	0.6309	0.6369	0.1091	0.3834	0.5298	0.5399	0.5873	0.6208	0.1019	0.3761	0.5222	0.6108	0.4856	0.5261	0.5447
21	22	rs6001084			38681644	A	0.1495	0.4	G	15.55	8.055E-05	0.2636	8.055E-05	9.884E-05	1	1	1	1	0.9301	1	no/alfa project						0.2058	0.20311	0.1697	0.1717	0.41	0.3	0.029			
Row 2-6 are the details of SNPs among 255 susceptible SNPs from literature search in our data																																				
Row 7-22 are the details of SNPs in GSA with p value 0.00001in our data																																				

Columns (1-21) are results obtained in our data set from GSA analysis.

Columns (22-37) are the information retrieved from the database: INDIGEN https://clingen.igib.res.in/indigen
